# Supplementary material for: Detection and evaluation of signals associated with exposure to individual and combination of medications in pregnancy: a signal detection study protocol
Source: BMJ Open. 2023 Oct 9;13(10):e073162. doi: 10.1136/bmjopen-2023-073162 (PMC10565241; doi:10.1136/bmjopen-2023-073162)
Supplement: Supplementary data [file bmjopen-2023-073162supp001.pdf]

Supplementary Table 1: Operational definition of pregnancy outcomes included in this signal detection study

Outcomes	Operational definition	Observation time window
**Miscarriage**	(1) Diagnostic code in primary care OR<br>(2) Diagnostic code in secondary care OR<br>(3) Database specific flag for miscarriage	Diagnostic code or flag recorded until 30 weeks of pregnancy (includes 6-week lag period for recording)
**Intrauterine death / Stillbirth / perinatal death**	(1) Diagnostic code in primary care OR<br>(2) Diagnostic code in secondary care OR<br>(3) Database specific flag for IUD/Stillbirth/perinatal death	Diagnostic code or flag recorded from 24 weeks of pregnancy until six weeks after delivery date (includes 6-week lag period for recording)
**Small for gestational age (SGA)**	(1) Diagnostic code in primary care OR<br>(2) Diagnostic code in secondary care OR<br>(3) Database specific flag for SGA OR<br>(4) Database specific continuous variable for gestational age, sex and birthweight (indicating birthweight below the 10th percentile for babies of the same gestational age according to the INTERGROWTH-21st birth weight standard)	Diagnostic code or flag recorded +/- 6 weeks from delivery date
**Preterm birth**	(1) Diagnostic code in primary care OR<br>(2) Diagnostic code in secondary care OR<br>(3) Database specific flag for preterm birth OR<br>(4) Database specific continuous variable for gestational age (indicating gestational age between 22 to 37 weeks)<br>(5)	Diagnostic code or flag recorded +/- 6 weeks from delivery date
**Overall Congenital anomalies (CA)**	(1) Diagnostic code in primary care OR<br>(2) Diagnostic /operational code in secondary care OR<br>(3) Database specific flag for congenital anomalies	Diagnostic code or flag recorded in the baby's medical records until 1 year after birth (delivery date)
**Specific major congenital anomalies**	(1) Diagnostic code in primary care OR<br>(2) Diagnostic code in secondary care OR<br>(3) Database specific flag for congenital anomalies	Diagnostic code or flag recorded in the baby's medical records until 1 year after birth (delivery date)
**Maternal death**	(1) Death flag in primary care OR<br>(2) Death flag in the linked death registry	Death flag recorded until 1 year after pregnancy end date

**Pre-eclampsia, eclampsia, HELLP syndrome, gestational hypertension**	(1) Diagnostic code in primary care OR<br>(2) New primary care prescription of antihypertensives during pregnancy OR<br>(3) Diagnostic code in secondary care OR<br>(4) Database specific flag for pre-eclampsia/HELLP syndrome/gestational hypertension<br><br>Exclusion: Patients with a pre-existing record of chronic hypertension or antihypertensives prior to pregnancy or within 20 weeks of pregnancy	Diagnostic code recorded from 24 weeks of pregnancy until six weeks after pregnancy end date (includes 6-week lag period for recording)
**Placenta abruption**	(1) Diagnostic code in primary care OR<br>(2) Diagnostic code in secondary care OR<br>(3) Database specific flag for placenta abruption	Diagnostic code or flag recorded until 6 weeks after pregnancy end date (includes 6-week lag period for recording)
**Venous thromboembolism**	(1) Diagnostic code in primary care OR<br>(2) Diagnostic code in secondary care OR<br>(3) Database specific flag for venous thromboembolism	Diagnostic code or flag recorded until 12 weeks after pregnancy end date (includes 6-week lag period for recording)
**Preterm premature rupture of membrane (PPROM)**	(1) (Diagnostic code for premature rupture of membrane in primary care OR<br>(2) Diagnostic code for premature rupture of membrane in secondary care OR<br>(3) Database specific flag for premature rupture of membrane)<br><br>AND<br><br>Operational definition met for premature birth	Diagnostic code or flag recorded +/- 6 weeks from delivery date
**Severe maternal morbidity**	(1) Diagnostic code in primary care OR<br>(2) Diagnostic /operational code in secondary care for one of the following morbidities<br><br>1. Acute myocardial infarction<br>2. Aneurysm<br>3. Acute Renal Failure<br>4. Adult respiratory distress syndrome<br>5. Amniotic fluid embolism<br>6. Cardiac arrest or ventricular fibrillation<br>7. Disseminated intravascular coagulation<br>8. Eclampsia	Diagnostic code recorded until 3 months after pregnancy end date

	9. Heart failure or arrest during surgery or procedure<br>10. Puerperal cerebrovascular disorders<br>11. Pulmonary oedema or Acute heart failure<br>12. Severe anaesthesia complications<br>13. Sepsis<br>14. Shock<br>15. Sickle cell disease with crisis<br>16. Air and thrombotic embolism<br>17. Conversion of cardiac rhythm<br>18. Blood products transfusion<br>19. Hysterectomy<br>20. Temporary tracheostomy<br>21. Ventilation	
**Postpartum haemorrhage (PPH)**	(1) Diagnostic code in primary care OR<br>(2) Diagnostic /operational code in secondary care	Diagnostic code recorded until 3 months after pregnancy end date
**Self-harm/suicide**	(1) Diagnostic code in primary care OR<br>(2) Diagnostic /operational code in secondary care OR<br>(3) Suicide as cause of death in the linked death registry	Diagnostic code or death flag recorded until 1 year after pregnancy end date
**Postpartum mental illness**	(1) Diagnostic code in primary care OR<br>(2) Diagnostic code in secondary care	Diagnostic code recorded until 1 year after pregnancy end date
**Cerebral palsy/Autism/ADHD/Neurodevelopmental outcomes**	(1) Diagnostic code in primary care OR<br>(2) Diagnostic code in secondary care	Diagnostic code recorded anytime in the baby's medical records

Supplementary Table 2: The RECORD statement – checklist of items, extended from the STROBE statement, that should be reported in observational studies using routinely collected health data.

	Item No.	STROBE items	Location in manuscript where items are reported	RECORD items	Location in manuscript where items are reported
**Title and abstract**					
	1	(a) Indicate the study's design with a commonly used term in the title or the abstract (b) Provide in the abstract an informative and balanced summary of what was done and what was found		RECORD 1.1: The type of data used should be specified in the title or abstract. When possible, the name of the databases used should be included.<br><br>RECORD 1.2: If applicable, the geographic region and timeframe within which the study took place should be reported in the title or abstract.<br><br>RECORD 1.3: If linkage between databases was conducted for the study, this should be clearly stated in the title or abstract.	Multiple databases are planned to be used. This is mentioned in methods and analysis section of the abstract.<br><br>Mentioned in the methods and analysis section of the abstract.<br><br>No linkage involved in the study
**Introduction**					
Background rationale	2	Explain the scientific background and rationale for the investigation being reported			Introduction section, para 4
Objectives	3	State specific objectives, including any prespecified hypotheses			Aims section
**Methods**					
Study Design	4	Present key elements of study design early in the paper			Study design section within

					methods and analysis
Setting	5	Describe the setting, locations, and relevant dates, including periods of recruitment, exposure, follow-up, and data collection			Table 1
Participants	6	*(a) Cohort study* - Give the eligibility criteria, and the sources and methods of selection of participants. Describe methods of follow-up<br>*Case-control study* - Give the eligibility criteria, and the sources and methods of case ascertainment and control selection. Give the rationale for the choice of cases and controls<br>*Cross-sectional study* - Give the eligibility criteria, and the sources and methods of selection of participants<br><br>*(b) Cohort study* - For matched studies, give matching criteria and number of exposed and unexposed<br>*Case-control study* - For matched studies, give matching criteria and the number of controls per case		RECORD 6.1: The methods of study population selection (such as codes or algorithms used to identify subjects) should be listed in detail. If this is not possible, an explanation should be provided.<br><br>RECORD 6.2: Any validation studies of the codes or algorithms used to select the population should be referenced. If validation was conducted for this study and not published elsewhere, detailed methods and results should be provided.<br><br>RECORD 6.3: If the study involved linkage of databases, consider use of a flow diagram or other graphical display to demonstrate the data linkage process, including the number of individuals with linked data at each stage.	Table 1<br><br>Reference number 23<br><br>No linkage
Variables	7	Clearly define all outcomes, exposures, predictors, potential confounders, and effect modifiers. Give diagnostic criteria, if applicable.		RECORD 7.1: A complete list of codes and algorithms used to classify exposures, outcomes, confounders, and effect modifiers should be provided. If these cannot be reported, an explanation should be provided.	This is a signal detection study with a wide range of exposures and outcomes.
Data sources/ measurement	8	For each variable of interest, give sources of data and details			Table 2

		of methods of assessment (measurement).<br>Describe comparability of assessment methods if there is more than one group			
Bias	9	Describe any efforts to address potential sources of bias			Systematic signal review section under methods and analysis
Study size	10	Explain how the study size was arrived at			Table 1
Quantitative variables	11	Explain how quantitative variables were handled in the analyses. If applicable, describe which groupings were chosen, and why			Exposure section, paragraph 2 and covariates section under methods and analysis
Statistical methods	12	(a) Describe all statistical methods, including those used to control for confounding<br>(b) Describe any methods used to examine subgroups and interactions<br>(c) Explain how missing data were addressed<br>(d) *Cohort study* - If applicable, explain how loss to follow-up was addressed<br>*Case-control study* - If applicable, explain how matching of cases and controls was addressed<br>*Cross-sectional study* - If applicable, describe analytical methods taking account of sampling strategy			(a) Statistical Analysis section, para 2 under methods and analysis<br>(b) No subgroups<br>(c) Covariates section under methods and analysis<br>(d) No matching

		(e) Describe any sensitivity analyses			(e) No sensitivity analyses
Data access and cleaning methods		..		RECORD 12.1: Authors should describe the extent to which the investigators had access to the database population used to create the study population.<br><br>RECORD 12.2: Authors should provide information on the data cleaning methods used in the study.	Table 1<br><br>Described elsewhere (Reference 23)
Linkage		..		RECORD 12.3: State whether the study included person-level, institutional-level, or other data linkage across two or more databases. The methods of linkage and methods of linkage quality evaluation should be provided.	No linkage
**Results**					
Participants	13	(a) Report the numbers of individuals at each stage of the study (*e.g.*, numbers potentially eligible, examined for eligibility, confirmed eligible, included in the study, completing follow-up, and analysed)<br>(b) Give reasons for non-participation at each stage.<br>(c) Consider use of a flow diagram		RECORD 13.1: Describe in detail the selection of the persons included in the study (*i.e.,* study population selection) including filtering based on data quality, data availability and linkage. The selection of included persons can be described in the text and/or by means of the study flow diagram.	Study population section under methods and analysis
Descriptive data	14	(a) Give characteristics of study participants (*e.g.*, demographic, clinical, social) and information on exposures and potential confounders<br>(b) Indicate the number of participants with missing data for each variable of interest			This is a protocol paper

		(c) *Cohort study* - summarise follow-up time (*e.g.*, average and total amount)			
Outcome data	15	*Cohort study* - Report numbers of outcome events or summary measures over time<br>*Case-control study* - Report numbers in each exposure category, or summary measures of exposure<br>*Cross-sectional study* - Report numbers of outcome events or summary measures			This is a protocol paper
Main results	16	(a) Give unadjusted estimates and, if applicable, confounder-adjusted estimates and their precision (e.g., 95% confidence interval). Make clear which confounders were adjusted for and why they were included<br>(b) Report category boundaries when continuous variables were categorized<br>(c) If relevant, consider translating estimates of relative risk into absolute risk for a meaningful time period			This is a protocol paper
Other analyses	17	Report other analyses done—e.g., analyses of subgroups and interactions, and sensitivity analyses			This is a protocol paper
**Discussion**					
Key results	18	Summarise key results with reference to study objectives			This is a protocol paper
Limitations	19	Discuss limitations of the study, taking into account sources of potential bias or imprecision.		RECORD 19.1: Discuss the implications of using data that were not created or collected to answer the specific research question(s). Include	Systematic Signal review section under methods and analysis

		Discuss both direction and magnitude of any potential bias		discussion of misclassification bias, unmeasured confounding, missing data, and changing eligibility over time, as they pertain to the study being reported.	
Interpretation	20	Give a cautious overall interpretation of results considering objectives, limitations, multiplicity of analyses, results from similar studies, and other relevant evidence			This is a protocol paper
Generalisability	21	Discuss the generalisability (external validity) of the study results			This is a protocol paper
**Other Information**					
Funding	22	Give the source of funding and the role of the funders for the present study and, if applicable, for the original study on which the present article is based			Funding section
Accessibility of protocol, raw data, and programming code		..		RECORD 22.1: Authors should provide information on how to access any supplemental information such as the study protocol, raw data, or programming code.	This is a protocol paper

*Reference: Benchimol EI, Smeeth L, Guttmann A, Harron K, Moher D, Petersen I, Sørensen HT, von Elm E, Langan SM, the RECORD Working Committee. The REporting of studies Conducted using Observational Routinely-collected health Data (RECORD) Statement. *PLoS Medicine* 2015; in press.

*Checklist is protected under Creative Commons Attribution (CC BY) license.
