# Supplementary material for: Detection and evaluation of signals associated with exposure to individual and combination of medications in pregnancy: a signal detection study protocol
Source: BMJ Open. 2023 Oct 9;13(10):e073162. doi: 10.1136/bmjopen-2023-073162 (PMC10565241; doi:10.1136/bmjopen-2023-073162)
Supplement: Author's manuscript [file bmjopen-2023-073162.draft_revisions.pdf]

BMJ Open

BMJ Open is committed to open peer review. As part of this commitment we make the peer review history of every article we publish publicly available.

When an article is published we post the peer reviewers' comments and the authors' responses online. We also post the versions of the paper that were used during peer review. These are the versions that the peer review comments apply to.

The versions of the paper that follow are the versions that were submitted during the peer review process. They are not the versions of record or the final published versions. They should not be cited or distributed as the published version of this manuscript.

BMJ Open is an open access journal and the full, final, typeset and author-corrected version of record of the manuscript is available on our site with no access controls, subscription charges or pay-per-view fees (http://bmjopen.bmj.com).

If you have any questions on BMJ Open's open peer review process please email info.bmjopen@bmj.com

BMJ Open

Detection and evaluation of signals associated with exposure to individual and combination of medications in pregnancy: A signal detection study protocol

Journal:	*BMJ Open*
Manuscript ID	bmjopen-2023-073162
Article Type:	Protocol
Date Submitted by the Author:	21-Mar-2023
Complete List of Authors:	Subramanian, Anuradhaa; University of Birmingham, Institute of Applied Health Research<br>Lee, Siang Ing; University of Birmingham Institute of Applied Health Research<br>Hemali Sudasinghe, Sudasing Pathirannehelage Buddhika; University of Birmingham, Institute of Applied Health Research<br>Wambua, Steven; University of Birmingham, Applied health research<br>Phillips, Katherine; University of Birmingham, Applied health research<br>Singh, Megha; University of Birmingham, Applied health research<br>Azcoaga-Lorenzo, Amaya; University of Saint Andrews School of Medicine, Division of Population and Behavioural Sciences; Instituto de Investigación Sanitaria Fundación Jimenez Diaz, Hospital Rey Juan Carlos. Research Network on Chronicity, Primary Care and Health Promotion-RICAPPS (RICORS)<br>Cockburn, Neil; University of Birmingham, Applied health research<br>Wang, Jingya; Institute of Applied Health Research University of Birmingham<br>Fagbamigbe, Adeniyi; University of St Andrews, PBS; University of Ibadan, EMS<br>Usman, Muhammad; University of Ibadan, University of St Andrews<br>Damase-Michel, Christine; Service de Pharmacologie Médicale, CHU Toulouse, Université Toulouse III<br>Yau, Christopher; The University of Manchester, Division of Informatics, Imaging and Data Sciences, Faculty of Biology Medicine and Health<br>Kent, Lisa; Queen's University Belfast, Centre for Public Health<br>McCowan, Colin ; University of St. Andrews<br>OReilly, Dermot; Queens University Belfast, Epidemiology and Public Health<br>Santorelli, Gillian; Bradford Teaching Hospitals NHS Foundation Trust<br>Hope, Holly; Manchester University<br>Kennedy, Jonathan; Swansea University, College of Medicine<br>Mhereeg, Mohamed; Swansea University Medical School<br>Abel, Kathryn ; Institute of Brain Behaviour and Mental Health, Medical and Human Sciences; Manchester Mental Health & Social Care Trust<br>Eastwood, Kelly-Ann; University Hospitals Bristol and Weston NHS Foundation Trust<br>Black, Mairead; University of Aberdeen<br>Loane , Maria ; University of Ulster<br>Moss, Ngawai; Queen Mary University of London

Brophy, Sinead; Swansea University, College of Medicine
Brocklehurst, Peter; University of Birmingham
Dolk, Helen; Ulster University - Jordanstown Campus, Institute for Nursing and Health Research
Nelson-Piercy, Catherine; Guy’s & St Thomas’ Foundation Trust
Nirantharakumar, Krishnarajah; University of Birmingham

Keywords:	OBSTETRICS, Maternal medicine < OBSTETRICS, EPIDEMIOLOGY

SCHOLARONE™
Manuscripts

Detection and evaluation of signals associated with exposure to individual and combination of medications in pregnancy: A signal detection study protocol

Authors: Anuradhaa Subramanian[1], Siang Ing Lee[1], Sudasing Pathirannehelage Buddhika Hemali Sudasinghe[1], Steven Wambua[1], Katherine Phillips[1], Megha Singh[1], Amaya Azcoaga-Lorenzo[2,3], Neil Cockburn[1], Jingya Wang[1], Adeniyi Francis Fagbamigbe[2,4], Muhammad Usman[3], Christine Damase-Michel[5,6], Christopher Yau[7,8], Lisa Kent[9], Colin McCowan[2], Dermot O'Reilly[9], Gillian Santorelli[10], Holly Hope[11], Jonathan I Kennedy[12], Mohamed Mhereeg[12], Kathryn M Abel[11,13], Kelly-Ann Eastwood[9, 14], Mairead Black[15], Maria Loane[16], Ngawai Moss[17], Sinead Brophy[12], Peter Brocklehurst[1#], Helen Dolk[18#], Catherine Nelson-Piercy[19#], Krishnarajah Nirantharakumar[1#], on behalf of the MuM-PreDiCT Group.

[#] joint senior authors

Corresponding author: Prof Krishnarajah Nirantharakumar
k.nirantharan@bham.ac.uk

Affiliation:

1. Institute of Applied Health Research, University of Birmingham, Birmingham, UK
2. Division of Population and Behavioural Sciences, School of Medicine, University of St Andrews, UK
3. Hospital Rey Juan Carlos, Instituto Investigacion Sanitaria Fundacion Jimenez Diaz
4. Department of Epidemiology and Medical Statistics, College of Medicine, University of Ibadan, Nigeria
5. Medical and Clinical Pharmacology, School of Medicine, Université Toulouse III, France
6. INSERM, Center for Epidemiology and Research in Population Health (CERPOP), France
7. Division of Informatics, Imaging and Data Sciences, Faculty of Biology Medicine and Health, The University of Manchester, UK
8. Health Data Research UK
9. Centre for Public Health, Queen's University of Belfast, UK
10. Bradford Institute for Health Research, UK
11. Centre for Women's Mental Health, Division of Psychology and Mental Health, School of Health Sciences, Faculty of Biology Medicine & Health, The University of Manchester, UK
12. Data Science, Medical School, Swansea University, UK
13. Greater Manchester Mental Health NHS Foundation Trust, Manchester, UK
14. St Michael's Hospital, University Hospitals Bristol NHS Foundation Trust, UK
15. Aberdeen Centre for Women's Health Research, School of Medicine, Medical Science and Nutrition, University of Aberdeen, UK
16. The Institute of Nursing and Health Research, Ulster University, UK
17. Patient and public representative, London, UK
18. Centre for Maternal, Fetal and Infant Research, The Institute of Nursing and Health Research, Ulster University, Coleraine, UK.

For peer review only

19. Guy's and St. Thomas' NHS Foundation Trust, UK

Abstract

Introduction

Considering the high prevalence of polypharmacy in pregnant women and the knowledge gap in the risk-benefit safety profile of their often-complex drug regimens, more research is needed to optimise prescribing. In this study, we aim to detect adverse and protective effect signals of exposure to individual and pairwise combinations of medications during pregnancy.

Methods and analysis

Using a range of real-world data sources from all four nations of the UK, we aim to conduct a pharmacovigilance study to assess the safety of medications prescribed during the preconception period (three months prior to conception) and first trimester of pregnancy. Women aged between 15-49 years with a record of pregnancy within the Clinical Practice Research Datalink (CPRD) Pregnancy Register, the Welsh Secure Anonymised Information Linkage (SAIL), the Scottish Morbidity Record datasets (SMR), and the Northern Ireland Maternity System (NIMATS) will be included.

A series of case control studies will be conducted to estimate measures of disproportionality, detecting signals of association between a range of pregnancy, and exposure to individual and combinations of drugs.

A multidisciplinary expert team will be invited to a signal detection workshop. By employing a structured framework, signals will be transparently assessed by each member of the team using a questionnaire appraising the signals on aspects of temporality, selection, time and measurement related biases and confounding by underlying disease or co-medications. Through group discussion, the expert team will reach consensus on each of the drug-outcome signal, thereby excluding spurious signals, leaving signals suggestive of causal associations for further evaluation.

Ethics and dissemination

Ethical approval will be obtained from the Independent Scientific Advisory Committee, SAIL Information Governance Review Panel, University of St. Andrews Teaching and Research Ethics Committee and Office for Research Ethics Committees Northern Ireland (ORECNI) for access and use of CPRD, SAIL, SMR and NIMATS data respectively.

For peer review only

Introduction

Conventional methods of drug development from discovery to pre-clinical and clinical research and review are expensive, time consuming and disproportionately concentrate on disease areas that predominantly affect men.[1] The effect of this can be seen in the female dominance of adverse drug effect reporting, especially during the period of reproductive age.[2] Pregnant and breast-feeding women are further inclined to become `therapeutic orphans', by their routine exclusion from clinical trials, given the struggle to insure such trials involving pregnant women. Pregnant women are further disadvantaged by having to arduously navigate through multifarious social influences, some losing their autonomy in medical decision making around taking medications or in their choice to participate in a clinical trial during pregnancy.[3,4]

In light of the urgent action required to develop safe drugs for use during pregnancy, the 'Healthy Mum, Healthy Baby, Healthy Future' report by the Birmingham Health Partners advocates inclusion of pregnant women in clinical trials unless there are specific safety concerns.[5] The report also makes a case to pilot a 'Maternal Investigation Plan' similar to the 'Paediatric Investigation Plan' implemented by European Union, to make licensing compulsory for use of medications during pregnancy.

In the absence of safety signals for drug exposure during pregnancy from clinical trial data, information on teratogenicity is limited and is only prospectively collected from national surveillance data, available from resources such as UK Teratology Information Service (UKTIS).[6] This limited availability of robust evidence on drug use during pregnancy has led to women often being prescribed medications 'off-label' in practice.[7] Without appropriate drug safety information, the decision to continue, discontinue or switch their medication falls to the women themselves and their healthcare providers.[8]

We have previously found that the prevalence of polypharmacy in the UK, during the first trimester of pregnancy alone, has increased from 8.7% to 18.7% over the last two decades.[9] While polypharmacy itself may be essential and beneficial in the management of multiple chronic conditions in combination with pregnancy related complications, inappropriate polypharmacy may cause preventable adverse drug events (ADEs) due to drug-drug interactions.[10] Considering the high prevalence of polypharmacy among women of reproductive age and the knowledge gap in safety profiles of complex drug regimen,[9,11–13] more research is urgently needed to optimise prescribing by detecting signals of adverse outcomes following exposure to combined medications.

Real world data (RWD) can be used to assess the post-market safety of individual and combined medication use during pregnancy, generating evidence to support regulatory decision making. In addition, RWD also provides an opportunity for repurposing of approved medications as prophylactic treatments for pregnancy related complications.[14] Real world data-based signal detection involves a systematic step-by-step approach[15–17]: (1) identifying real world data source and inspecting its feasibility in accurately ascertaining exposures and outcomes to support identification of safety signals, (2) listing the outcomes (adverse events) and exposures (medications) of interest, (3) generating measures of statistical association for large sets of exposure-outcome pairs with methodological design to limit confounding, (4) reviewing identified signals by a multidisciplinary team to ensure contextual interpretation, and consider sources of bias that lead to false positive signals, and (5) strengthening and confirming screened signals using rigorous pharmaco-epidemiological studies or clinical trials.

For peer review only

The feasibility of mining real world data to detect adverse and protective effect signals of individual drugs during pregnancy has already been established.[16,18] In this study, as part of the "Multimorbidity and Pregnancy: Determinants, Clusters, Consequences and Trajectories (MuM-PreDiCT)" consortium, we aim to develop and implement a systematic signal detection methodology to determine adverse and protective signals from both individual and combinatorial drug exposure on the incidence of various pregnancy outcomes, using a set of large primary care, secondary care and maternity care databases within the UK.

Aims

The study aims to evaluate the feasibility of conducting a signal detection study estimating and evaluating the adverse and protective signals of medications prescribed during the preconception period and the first trimester of pregnancy on the incidence of various pregnancy outcomes using real world data

The key objectives include:

(1) To scope real world data sources in UK and check their feasibility for signal detection by exploring the range of medications prescribed and recorded within said data sources during the preconception period and first trimester of pregnancy
(2) To identify a core list of pregnancy outcomes and explore their feasibility in terms of the recorded prevalence of these outcomes within said data sources
(3) To apply suitably developed methods from published literature to estimate measures of disproportionality for each of the exposure-outcome pairs and detect signals
(4) To systematically review the detected signals through a multidisciplinary expert committee workshop, using a pre-specified structured questionnaire exploring sources of false positives.

Methods and analysis

Data source

Four population-based data sources spanning all four nations of the United Kingdom (UK) will provide data for this signal detection study. These include primary care data sources such as Clinical Practice Research Datalink (CPRD, from all four nations) and The Secure Anonymized Information Linkage (SAIL, Wales) and secondary care data sources such as Hospital Episode Statistics (HES, England), Scottish Morbidity Records (SMR) with linked community prescription data (Scotland) and Northern Ireland Maternity System (NIMATS) with linked Enhanced Prescribing Database (EPD, Northern Ireland). The data sources are scoped and their feasibility for this signal detection study is assessed and tabulated in Table 1-4.

For peer review only - http://bmjopen.bmj.com/site/about/guidelines.xhtml

(1) Clinical Practice Research Datalink (CPRD) and Hospital Episode Statistics (HES)

CPRD Gold and Aurum contains anonymized, longitudinal medical records of over 20 and 39 million patients in the UK collected by over 985 and 1,489 participating general practices respectively as part of their care and support.[19] It currently covers general practices that uses the Vision and EMIS software, and collects data from 20% of general practices in the UK.[20] It includes data on demographics, diagnoses and prescriptions. Linkage to area-based deprivation index known as the Index of Multiple Deprivation (IMD) and Hospital Episodes Statistics (HES) is available for 75% of patients in England, whose general practices have consented to the CPRD linkage scheme.

CPRD Gold contains data on medications prescribed within primary care with associated prescription time stamps, encoded using drug codes that are assigned and categorized according to British National Formulary (BNF) item codes. However, CPRD Gold may be limited by their unavailability of secondary care and over-the-counter prescription data and data on whether these prescriptions were dispensed.

Within CPRD, the CPRD Pregnancy Register is an algorithm that takes information from maternity, antenatal and birth health records from primary care to detect pregnancy episodes and their outcomes.[21] The Pregnancy register consolidates and provides details of unique pregnancy episodes, their dates and outcomes. A subset of the pregnancy register, Mother Baby Linked (MBL) data further allows for studying outcomes in the children.

(2) The *Secure Anonymized Information Linkage (SAIL)*

The SAIL databank, a population level database in Wales, is a repository of anonymized health and socio-economic administrative data that provides linkage at an individual level.[22] It holds health data for 4.8 million people and includes data contributed by 80% of Welsh GP practices. Within SAIL, the National Community Child Health Dataset, GP and hospital records have previously been used to detect pregnancies.[23] In addition, patient level linkage to the Welsh Longitudinal General Practice dataset and the Welsh Demographic Service dataset have been used to obtain data on diagnoses, prescriptions and demographics data respectively. The Welsh Dispensing Data Set (WDDS) containing information on GP prescribed medications, dispensed medications by community contractors have been linked to the SAIL databank. Similar to CPRD, SAIL may be limited by their unavailability of secondary care and over-the-counter prescription data and data on whether the prescriptions were dispensed.

(3) Scottish Maternity Records and linked data sources

The Scottish Maternity Records (SMR02) will be linked to data from Hospital Admissions (SMR01), Mental Health Inpatients (SMR04), Accident and Emergency, and the Demography and Death registry,[23] covering diagnoses and demographic data for all inpatient stays and day cases for residents in Scotland. Maternity records (SMR02) or pregnancy-related hospital admissions (SMR01) allows for identification of pregnancies, and prescription data of medications dispensed in the community can be obtained from linked Prescribing Information System (PIS).

For peer review only

(4) Northern Ireland Maternity System (NIMATs)

NIMATs holds demographic and clinical information on mothers and infants. It captures data relating to the current complete maternity process, but also contains details about the mother's past medical and obstetric history. NIMATs contains information on medications that mothers self-reported to have taken during pregnancy. In addition to self-reported data on medications, linkage of NIMATs to EPD allows for analysis of prescription data issued by GPs.[24]

Study design

A series of case control studies will be conducted to estimate measures of disproportionality, detecting signals of association between each of the pregnancy outcomes of interest, and exposure to individual or combination of drugs prescribed during the preconception period and first trimester of pregnancy.

Study population

For this feasibility study, we aim to conduct the analyses separately across all four databases described in the section above. Women aged between 15-49 years with a pregnancy recorded within the CPRD Gold Pregnancy Register (all four nations), National Community Child Health data (Wales), SMR02 (Scotland) and NIMATS (Northern Ireland), within the study period customized to the period of data availability within each data source will be eligible for inclusion in this study (Table 1-4). Data standard quality checks for each of the database and eligibility criteria for inclusion is presented in a previous publication.[23] Pregnancy start dates will be either derived using a pre-defined algorithm or used as is reported within the said data source (Table 1-4) and will be used to define exposure and outcome time window.

Outcomes

The MuM-PreDiCT and ConcePTION consortium have developed core outcome sets (a minimum set of recommended maternal and offspring outcomes) for studies of pregnant women with multiple long-term conditions, and for studies generating medication safety evidence respectively.[25,26] The outcome set was reviewed by a study advisory group panel comprising of general practitioners, obstetricians, obstetric physicians and experienced users of the available data sources, to identify the pregnancy outcomes' suitability and feasibility for inclusion in this signal detection study. The availability, prevalence, quality, and completeness of data recording of these outcomes within the said data sources were used as criteria to determine the feasibility of the outcome of interest in this signal detection study. Outcomes that cannot be considered as a medication adverse event such as termination of pregnancy, outcomes that were too broad or non-specific such as involvement of patients in care decisions, and neonatal outcomes that were too were too narrow and reflective of prematurity such a intubation/ventilation requirement were excluded. The final list of outcomes to be included is available in Table 5.

Exposure

All medications prescribed within primary care [as in the CPRD Gold database (all four nations) and EPD (Northern Ireland)] and all medications dispensed in the community [as in the PIS database (Scotland)] have a BNF item code associated with them in the UK.

For peer review only

Dispensed medications in WDDS (Wales) are coded based on the Dictionary of Medicines and Devices (DM+D). Using a complete extract of the dictionary,[27] each dispensed item within the dictionary can be mapped to a corresponding BNF Item code.

Analysis stratified by BNF codes has been established in a previous analysis, wherein using a dictionary of drugs prescribed within primary care (recorded in CPRD Gold), we stratified the medications prescribed according to their 4-digit BNF Item code (BNF chapter, section, paragraph and sub-paragraph), screened and selected 577 BNF items that were pharmacological agents with therapeutic action.[9]

Using a similar strategy, we will ascertain the exposure information for a range of drugs stratified by their BNF Item code specifically within two crucial time windows: (1) preconception period (up to 90 days prior to the start of pregnancy), and (2) first trimester of pregnancy (first twelve weeks of pregnancy). [28]

Furthermore, we will ascertain the exposure information for a range of drug pairs that are prescribed concurrently within the same exposure window to assess adverse and protective effect signals associated with combined medications.

While the data sources comprehensively capture medicines prescribed within primary care and within the community, the study is limited to medicines marketed and prescribed in the UK. Furthermore, signals from over-the-counter medications cannot be captured within this study.

Covariates

The following demographic and health characteristics will be obtained from the four datasets; maternal age at the start of pregnancy, ethnicity, smoking status as recorded prior to the start of pregnancy, pre-gravid body mass index (BMI) and a wide range of comorbidities. Ethnicity will be categorized as White, Asian, Black Afro-Caribbean, mixed ethnic background, and other ethnic minority groups including Chinese. Latest BMI recorded prior to the start of pregnancy will be considered as pre-gravid BMI and will be categorized according to the WHO definition as underweight (<18.5 kg/m^2), healthy weight (18.5-25 kg/m^2), overweight (25-30 kg/m^2) and obese (≥30 kg/m^2). Smoking status of patients prior to the start of pregnancy will categorise patients as current smokers, ex-smokers and never-smokers. Patients with missing data on smoking status, pre-gravid BMI and ethnicity will be categorized into a separate missing category within the corresponding variable. The list of 79 pre-existing long-term comorbidities for which baseline data were extracted and their definitions is presented in a previous publication. [23]

Statistical analysis

Description of baseline characteristics

Patient covariates will be summarized and stratified by their outcome status using number and percentages for categorical variables and mean (Standard Deviation) or median (Interquartile Range) for continuous variables.

Statistical analysis

Odds ratio will be considered as the primary measure of disproportionality and will be estimated for each of the exposure-outcome pairs using a series of univariate logistic regression models. In a series of adjusted logistic regression models, the exposure-outcome relationships will be adjusted for covariates including age at start of pregnancy, pre-gravid BMI, ethnicity, smoking status, and a disease risk score (DRS) to obtain adjusted odds ratios with 95% confidence intervals. DRS will be generated for each of the outcomes using logistic regression models considering the outcome as a dependent variable, and a range of pre-existing long-term conditions as independent variables. This is done to limit the effect of confounding attributable to prescriptions issued in order to manage the underlying long-term conditions. Relative decrease in p-value (p-RD) prior to and after adjustment for covariates will be calculated using the formula below.[16]

$$p - RD = \frac{p\ (before\ adj\ for\ DRS) - p(after\ adj\ for\ DRS)}{p\ (before\ adjustment)}$$

In addition to statistical measures of disproportionality, descriptive measures of numbers and proportions of eligible pregnancies with a prescription of the individual exposure drug or drug exposure combinations (pairs) during the two separate exposure windows will be presented stratified by their outcome status.

Benjamini–Hochberg correction for multiple testing will be applied with a threshold of 0.20.

The analyses in each of the four datasets will be conducted separately. Signals arising from each of the four data sources will be reviewed side-by-side through a systematic signal review workshop described below.

Systematic Signal review

A multidisciplinary expert team comprising of epidemiologists, general practitioners, obstetricians, obstetric physicians, pharmacists, data scientists, experts in genetics, internal medicine and pharmacovigilance, and researchers with expertise on the outcome of interest will be invited to a signal detection workshop. The workshop is aimed at collaboratively working and screening potential signals to identify and exclude signals that are likely to be affected by bias and confounding, leaving signals suggestive of causal associations to be further evaluated in future studies.

A list of all the exposure-outcome pairs, the adjusted and unadjusted OR along with 95% confidence intervals, and p-RD will be presented to the multidisciplinary team in the order of statistical significance and strength of association (high and low ORs). Exposure-outcome pairs that are marginally statistically insignificant will also be provided to the review team to avoid false negatives.

A checklist with the following items will be provided to the review team: (1) confirmation of exposure – outcome temporality to limit the possibility of reverse causality,[29] (2) consideration of concomitant medications to limit the possibility of co-prescription bias,[30] (3) consideration medical history, (4) consideration of demographic features, and (5) consideration of underlying disease and other alternative explanations to limit the possibility of confounding. After consideration of the checklist items, we will request each reviewer to mark the exposure-outcome pairs with three possible response options: (1) "already established", (2) "warrant

further investigation" and, (3) "dismissed", with reasons for each response. In a group discussion, conflicting responses will be discussed, and a consensus will be made.

Ethics approval and consent to participate

CPRD has ethics approval from the Health Research Authority to support research using anonymised patient data. A CPRD and linked HES data specific protocol will be written and submitted for approval to the Independent Scientific Advisory Committee. A SAIL databank specific protocol will be written and submitted for approval to the SAIL Information Governance Review Panel. For the use of SMR data, ethical approval will be requested from the School of Medicine Ethics Committee, acting on behalf of the University of St. Andrews Teaching and Research Ethics Committee. SAIL and SMR data will be analysed within a Safe Haven Research environment. For the use of NIMATS and EPS data, approval will be obtained from Office for Research Ethics Committees Northern Ireland (ORECNI) and the Honest Broker Governance Board.

Consent for publication

As the study data will be de-identified, consent for publication is not required.

Competing interests

None declared.

Funding statement

This work is independent research funded by the Strategic Priority Fund "Tackling multimorbidity at scale" programme (grant number MR/W014432/1) delivered by the Medical Research Council and the National Institute for Health Research in partnership with the Economic and Social Research Council and in collaboration with the Engineering and Physical Sciences Research Council. The views expressed are those of the author and not necessarily those of the funders, the NIHR or the UK Department of Health and Social Care. The funders had no role in study design, data collection and analysis, decision to publish, or preparation of the manuscript.

Patient and Public Involvement

Patient and public involvement (PPI) have been extensive from design to dissemination of research outputs from the MuM-PreDiCT group. Our PPI representatives have provided advice on the importance and relevance of this study and helped shape the research question. PPI representatives were also involved in screening and identifying the pregnancy outcomes of interest that are relevant to this study. One of the PPI representatives (NM) also co-authored this protocol. PPI representatives will be involved in the interpretation of the results in the future.

For peer review only

Table 1: Scoping real world data source from England to check its' feasibility for signal detection

Data	Features of the dataset	
CPRD	**Setting**	Primary care practices using the Vision® software system
	**Geographical region**	All four nations of the UK, with relatively few practices heavily concentrated in three conurbations and the South
	**Coverage**	4% of the UK
	**Linkage availability to**	• Pregnancy Register and Mother-Baby Linked data<br>• Deprivation data (Patient postcode linked deprivation measures, Practice postcode linked deprivation measures, Index of Multiple Deprivation (IMD), Townsend Deprivation Index, Carstairs Index, Rural-Urban Classification)<br>• Office of National Statistics (ONS) Death Registration Data<br>• Hospital Episode Statistics (HES)<br>• Cancer Registration Data
	**Data content (with linkage)**	• Patient demographics<br>• Consultation details<br>• Symptoms, signs and diagnoses<br>• Referrals to external care<br>• Immunisation details<br>• Records of test data in the GP system<br>• All prescriptions (drugs and appliances) issued by the GP
	**Period of data availability**	January 2000 to July 2022
	**Feasibility to identify pregnancy episodes?**	Yes, using CPRD Pregnancy Register
	**Sample size – number of pregnancy episodes**	1.5 million pregnancies

For peer review only

	**Definition of pregnancy start date**	As derived within the CPRD Pregnancy register
	**Feasibility to identify of exposures?**	Yes, using prescription records on the GP system. This is coded using the Dictionary of Medicines and Devices (DM+D). Each product within the dictionary is associated with a BNF code to which the product belongs. The range of exposures for this study will be defined using the BNF code.<br><br>Limitation: The data relates to primary care prescribing only. Secondary care prescribing data, prescriptions which are issued but never dispensed, over-the-counter prescriptions, and data on adherence to treatments are unavailable using this data source
	**Feasibility to identify of pregnancy outcomes?**	Yes, using Read code diagnoses within primary care or ICD-10 diagnoses within linked HES data.

For peer review only

Table 2: Scoping real world data source from Wales to check its' feasibility for signal detection

Data	Features of the dataset	
SAIL	**Setting**	A population level database in Wales. SAIL is a repository of anonymized health and socio-economic administrative data that provides linkage at an individual level using Trusted Third Party (TTP) in the NHS Wales Informatics Service (NWIS).
	**Geographical region**	Wales
	**Coverage**	The database contains 90% of all the GP data in Wales and 100% of all hospital admissions (PEDW)
	**Linkage availability to**	• Primary care data (from Wales Longitudinal General Practice (WLGP))<br>• Secondary care data (from inpatient hospital admissions, inpatient from Patient Episode Database for Wales (PEDW) and outpatient from Outpatient Database for Wales (OPDW)<br>• The National Community Child Health (NCCH)<br>• The Maternal Indicators (MIDS)<br>• The Welsh Dispensing Data Set (WDDS)<br>• The Welsh Demographic Service Dataset (WDSD)<br>• Office of National Statistics (ONS) Annual District Birth Extract<br>• Office of National Statistics (ONS) Annual District Death Extract<br>• Congenital Anomaly Register and Information Service (CARIS)
	**Data content (with linkage)**	• (WLGP): Signs, symptoms, test results, diagnoses, prescribed treatment, referrals for specialist treatment<br>• (PEDW): attendance and clinical information, diagnoses and operations performed. (OPDW): attendance information for all NHS Wales hospital outpatient appointments<br>• (NCCHD): Comprises information pertaining to birth registration, monitoring of child health examinations, and immunisations.<br>• (MIDS): contains data relating to the woman at initial assessment and to the mother and baby (or babies) for all births.

		• (WDDS): containing information on GP prescribed medications, dispensed medications by community contractors have been linked to the SAIL databank.<br>• (WDSD): used to extract Lower-layer Super Output Area (LSOA) version 2011 information associated with area-level deprivation (the Welsh Index for Multiple Deprivation (WIMD) version 2019)<br>• All births in Wales collected from birth registrations.<br>• All deaths relating to Welsh residents, including those that died out of Wales<br>Information about foetuses or babies who has or is suspected of having a congenital anomaly,
	**Period of data availability**	2000-2022
	**Feasibility to identify pregnancy episodes?**	Yes, using GP, PEDW, NCCHD, and MIDS datasets
	**Sample size – number of pregnancy episodes**	~475,000 pregnancies (from the year 2000 onwards)
	**Definition of pregnancy start date**	Pregnant women can be identified as any woman who have pregnancy codes in the WLGP data, in hospital admissions (PEDW) for pregnancy, or mothers in the NCCH or MIDS data with the baby birth date (Pregnancy end date) and gestational age at birth available.
	**Feasibility to identify of exposures?**	Yes, data covers prescriptions that are prescribed in Wales by GP's (general medical practitioners) and non-medical prescribers that have prescribed on behalf of the GP practice, that are then dispensed in the community within Wales. The data includes all prescribed medicines, dressings and appliances that are dispensed each month. If a patient does not take a prescription to the pharmacy for dispensing, then the information will not be included in the dataset. Information includes - the number of prescription items dispensed by each community pharmacy in Wales, broken down by the GP practice in which they prescribed, and also the number of prescription items prescribed in each GP practice in Wales broken down by the pharmacy that dispensed those items.
	**Feasibility to identify of pregnancy outcomes?**	Yes, using Read code diagnoses within primary care or ICD-10 diagnoses within hospital admissions data

For peer review only

Table 3: Scoping real world data source from Scotland to check its' feasibility for signal detection

Data	Features of the dataset	
SMR	**Setting**	Scottish Morbidity Records (SMR) from secondary care data
	**Geographical region**	Scotland
	**Coverage**	All obstetric inpatients and day cases from maternity hospitals in Scotland
	**Linkage availability to**	• Community Prescriptions
	**Data content (with linkage)**	• SMR00 - Outpatients<br>• SMR01 – Inpatient and day cases<br>• SMR02 - Maternity<br>• SMR04 – Mental Health<br>• SMR06 – Cancer<br>• Accident & Emergency<br>• NRS Deaths<br>• Prescribing Information System (community)<br>• Scottish Birth Record (SBR)<br>• National Records of Scotland (NRS) Infant Deaths<br>• NRS Stillbirths
	**Period of data availability**	SMR02 (pregnancy identification) from 2008-2021 with historical data from women<br>SMR01, SMR00, PIS and A&E database for the first 5 years of life on the children
	**Feasibility to identify pregnancy episodes?**	Yes
	**Sample size – number of pregnancy episodes**	670, 811 births + pregnancies not ending in births
	**Definition of pregnancy start date**	Based on gestational age at booking appointment and or outcome of pregnancy

For peer review only

	**Feasibility to identify of exposures?**	Yes, using prescription from the community. This is coded using the Dictionary of Medicines and Devices (DM+D). Each product within the dictionary is associated with a BNF code to which the product belongs. The range of exposures for this study will be defined using the BNF code.<br><br>Limitation: The data relates to primary care prescribing only. Secondary care prescribing data, prescriptions which are issued but never dispensed, over-the-counter prescriptions, and data on adherence to treatments are unavailable using this data source
	**Feasibility to identify of pregnancy outcomes?**	Only secondary care

For peer review only

Table 4: Scoping real world data source from Northern Ireland to check its' feasibility for signal detection

Data	Features of the dataset	
NIMATS	**Setting**	NIMATs is available in all five Health Trust areas across Northern Ireland (NI), within each hospital providing maternity services (11 hospitals in total). Access to NIMATS is also available to midwives/clerical staff in various community clinics across NI to allow for booking appointments to be recorded.
	**Geographical region**	Northern Ireland
	**Coverage**	100% within Northern Ireland; full coverage from 2011
	**Linkage availability to**	• NHAIS (Health Register) –<br>○ GP Patient Registration Index<br>○ Household characteristics (e.g. single person households)<br>○ Linkage to GRO (– Maternal deaths; Infant deaths (may be under-represented due to inability to link some infants to health records via Health & Care Number));<br>○ Via postcode,<br>○ NI Multiple Deprivation Measure (NIMDM) – area level deprivation, usually supplied as quintile or decile<br>○ Settlement Band Classification for urban/rural classification (2005-2014) (Post-code used only by HBS staff to aid linkage; not available to researchers)<br>○ Land & Property Services – rateable value of property (another measure of deprivation)<br>• Enhanced Prescribing Database (EPD) - database includes information on all drugs that have been prescribed by General Practitioners and dispensed to patients in NI.<br>• Secondary care data – includes hospital episodes from The Patient Administration System (PAS) and ED attendance data.<br>• NI Cerebral Palsy Register

For peer review only

	**Data content (with linkage)**	• Demographic information<br>• Clinical information on mothers, pregnancies and infants – delivery, investigations, medications, hospital stays, postnatal complications<br>• Mother's past medical and obstetric history.
	**Period of data availability**	2011-2022
	**Feasibility to identify pregnancy episodes?**	Yes. the mother is identified by her unique Health & Care Number, but she has a different hospital maternity number for each pregnancy.
	**Sample size – number of pregnancy episodes**	~300,000 pregnancies
	**Definition of pregnancy start date**	Can be derived from NIMATS variable EDC based on ultrasound scan
	**Feasibility to identify of exposures?**	Linkage to EPD; NIMATS also records medications taken at booking interview (maternal self-report), however this is a free-text variable and prone to variations in spelling. It is also deemed potentially disclosive and therefore not released to the researcher. Upon request, HBS may be able to provide a "dummy" variable for medications of particular interest.
	**Feasibility to identify of pregnancy outcomes?**	Recorded in NIMATS

For peer review only

Table 5: List of pregnancy outcomes included in this signal detection study from the core outcome set published by the ConcePTION and MuM-PreDiCT consortium

**List of core outcomes from ConcePTION consortium**	**Source of outcome documentation**			
	**CPRD**	**SAIL**	**NIMATS**	**SMR**
Gestational age at birth (in weeks or days)	Data available as variable in CPRD Pregnancy Register and linked HES data	Data available in National Community Child Health (NCCH) dataset in weeks	Variable recorded in NIMATS	Data available in SMR02
Miscarriage	Data available as variable in CPRD Pregnancy Register	Data unavailable<br>Currently, within SAIL researchers are unable to account for terminations as these are classed as sensitive data and not currently accessible for research purposes	Variable recorded in NIMATS, but only if it has occurred after booking appointment (early miscarriages may be under-represented)	Data available in SMR02 when women are admitted to secondary care
Intrauterine death / Stillbirth / perinatal death	Data available as variable in CPRD Pregnancy Register and linked HES data	Data available as stillbirths flagged in NCCH and Annual District Birth Extract (ABDE) datasets	IUD = Yes<br>Stillbirth = Yes<br>Perinatal death = partial (some infant deaths will not be captured in NIMATS but may be picked up in General Register Office (GRO) or (Patient Administration System) PAS data	Data available in SMR02
Small for gestational age (SGA)	Birthweight, baby's sex and gestational age data available within CPRD Pregnancy Register and linked HES data, from which SGA can be derived	Can be derived from birthweight data available in ADBE and NCCH, gestational age available in the latter	Yes – Variable "Birth Centile" available 2016 onwards<br>If missing, can be derived from NIMATS variables (birthweight, sex and gestational age at delivery)	Can be derived from variables recorded in SMR02 (birthweight, sex and gestational age at delivery)
Preterm Birth	Gestational age available within CPRD Pregnancy Register from which preterm birth can be derived	Data available in NCCH	Can be derived from variables recorded in NIMATS (gestational age at delivery, EDC)	Can be derived from variables recorded in SMR02 (gestational age at delivery, EDC)
Overall Congenital anomalies (CA)	Data available in primary care data, and linkage to baby records identified from mother-baby linked (MBL) data	Data available from the Congenital Anomaly Register and Information Service (CARIS) database and the NCCH_SIG_COND dataset which is used to inform it.	Yes – NIMATS (variable =infant complications; value = "congenital abnormality")	Data unavailable
Specific major congenital anomalies including termination of pregnancy due to fetal anomaly	Congenital anomalies recorded with sufficient quality will be included. Data available in primary care data, and linkage to baby records identified from mother-baby linked (MBL) data	Data available in CARIS database	Data unavailable.<br>Data in hospital discharge records are not suitable for identifying specific congenital anomalies.	Data unavailable

For peer review only

Maternal death	Data available in primary care data	Data available from GP/Patient Episode Data for Wales (PEDW)/ADDE for death during childbirth	Data available in GRO	Data available in National Records of Scotland
**Additional list of core outcomes from MuM-PreDiCT consortium**				
**Maternal outcomes**				
**Clinical: antenatal**				
Pre-eclampsia, eclampsia, HELLP syndrome	Data available in primary care and linked HES data	Data available in primary care and linked PEDW data (if diagnosed)	Data available in the Delivery details description, Present Pregnancy Problems, and Induction reason variables in NIMATS, as well as diagnoses variable in Patient Administration system (PAS)	Recorded from chapter XV of ICD10 Pregnancy, Childbirth and the Puerperium, (O00-O99).
Placenta abruption	Data available in primary care and linked HES data	Data available in primary care and linked PEDW data (if diagnosed)	Data available in the Delivery details description, Indication for Caesarean section variables in NIMATS, as well as diagnoses variable in Patient Administration system (PAS)	Recorded from chapter XV of ICD10 Pregnancy, Childbirth and the Puerperium, (O00-O99).
Venous thromboembolism	Data available in primary care and linked HES data	Data available in primary care and linked PEDW data (if diagnosed)	Data available as postnatal complications variable in NIMATS and as a diagnoses variable in Patient Administration system (PAS)	Recorded from chapter XV of ICD10 Pregnancy, Childbirth and the Puerperium, (O00-O99).
**Clinical: peripartum**				
Preterm premature rupture of membrane	Data available in linked HES data	Data available in primary care and linked PEDW data (if recorded)	Data available as postnatal complications variable in NIMATS and as a diagnoses variable in Patient Administration system (PAS)	Recorded from chapter XV of ICD10 Pregnancy, Childbirth and the Puerperium, (O00-O99).
Severe maternal morbidity	Specific maternal morbidities recorded with sufficient quality will be included. Data available from primary care data	Data available in primary care and linked PEDW data (if recorded)	Data available as specific maternal morbidities in NIMATS	Recorded from chapter XV of ICD10 Pregnancy, Childbirth and the Puerperium, (O00-O99).
Postpartum haemorrhage	Data available in linked HES data	Data available in primary care and linked PEDW data (if recorded)	Data available as postnatal complications variable in NIMATS and as a diagnoses variable in Patient Administration system (PAS)	Recorded from chapter XV of ICD10 Pregnancy, Childbirth and the Puerperium, (O00-O99).
**Mental health**				
Self-harm/suicide	Data available in primary care and linked HES data	Data available in primary care, linked PEDW, and emergency admissions data	Data available in Self Harm and Suicide Ideation Register	Data available in National Records of Scotland
**Children's outcomes**				
Cerebral palsy	Data available in primary care data, and linkage to baby records identified from mother-baby linked (MBL) data	Data available in primary and linked PEDW data	Data available in NI Cerebral Palsy Register (NICPR)	Data unavailable

For peer review only

Neurodevelopmental conditions	Data available in primary care data, and linkage to baby records identified from mother-baby linked (MBL) data		Data available if linked to Child Health System data	Data unavailable

For peer review only

References

1. Sundari Ravindran, T. K., Teerawattananon, Y., Tannenbaum, C. & Vijayasingham, L. Making pharmaceutical research and regulation work for women. *BMJ* **371**, (2020).
2. Watson, S., Caster, O., Rochon, P. A. & den Ruijter, H. Reported adverse drug reactions in women and men: Aggregated evidence from globally collected individual case reports during half a century. *EClinicalMedicine* **17**, 100188 (2019).
3. Figueroa Gray, M., Hsu, C., Kiel, L. & Dublin, S. Getting through the day: A pilot qualitative study of U.S. women's experiences making decisions about anti-nausea medication during pregnancy. *BMC Pregnancy Childbirth* **18**, 1–8 (2018).
4. Ngure, K. *et al.* Perceived Social Influences on Women's Decisions to use Medications not Studied in Pregnancy. A Qualitative Ethical Analysis of Preexposure Prophylaxis Implementation Research in Kenya. *J. Empir. Res. Hum. Res. Ethics* (2021) doi:10.1177/15562646211012296.
5. UNIVERSITYoF BIRMINGHAM The Case for UK Leadership in the Development of Safe, Effective and Accessible Medicines for Use in Pregnancy. (2022).
6. UKTIS. http://www.uktis.org/.
7. Ren, Z., Bremer, A. A. & Pawlyk, A. C. Drug development research in pregnant and lactating women. *Am. J. Obstet. Gynecol.* **225**, 33–42 (2021).
8. Lynch, M. M. *et al.* Making Decisions About Medication Use During Pregnancy: Implications for Communication Strategies. *Matern. Child Health J.* **22**, 92 (2018).
9. Subramanian, A. *et al.* Polypharmacy during pregnancy and associated risk factors: a retrospective analysis of 577 medication exposures among 1.5 million pregnancies in the UK, 2000-2019. *BMC Med.* **21**, 21 (2023).
10. Rochon, P. A. *et al.* Polypharmacy, inappropriate prescribing, and deprescribing in older people: through a sex and gender lens. *Lancet Heal. Longev.* **2**, e290–e300 (2021).
11. Prevalence of polypharmacy in pregnancy | SAPC. https://sapc.ac.uk/conference/2021/abstract/prevalence-of-polypharmacy-pregnancy_p1-29-2.
12. Steinberg, J. R. *et al.* Analysis of Female Enrollment and Participant Sex by Burden of Disease in US Clinical Trials Between 2000 and 2020. *JAMA Netw. Open* **4**, e2113749–e2113749 (2021).
13. Kaufman, D. W., Kelly, J. P., Rosenberg, L., Anderson, T. E. & Mitchell, A. A. Recent Patterns of Medication Use in the Ambulatory Adult Population of the United States: The Slone Survey. *JAMA* **287**, 337–344 (2002).
14. Huybrechts, K. F., Bateman, B. T. & Hernández-Díaz, S. Use of real-world evidence from healthcare utilization data to evaluate drug safety during pregnancy. *Pharmacoepidemiol. Drug Saf.* **28**, 906–922 (2019).
15. Cavadino, A. *et al.* Signal Detection in EUROmediCAT: Identification and Evaluation of Medication-Congenital Anomaly Associations and Use of VigiBase as a Complementary Source of Reference. *Drug Saf.* **44**, 765–785 (2021).
16. Demailly, R., Escolano, S., Haramburu, F., Tubert-Bitter, P. & Ahmed, I. Identifying Drugs Inducing Prematurity by Mining Claims Data with High-Dimensional Confounder Score Strategies. *Drug Saf.* **43**, 549–559 (2020).
17. Cederholm, S. *et al.* Structured Assessment for Prospective Identification of Safety Signals in Electronic Medical Records: Evaluation in the Health Improvement Network. *Drug Saf.* **38**, 87 (2015).
18. Final Report Summary - EUROMEDICAT (EUROmediCAT: Safety of Medication

For peer review only

use in Pregnancy in Relation to Risk of Congenital Malformations) | FP7 | CORDIS | European Commission. https://cordis.europa.eu/project/id/260598/reporting.
19. Herrett, E. *et al.* Data Resource Profile: Clinical Practice Research Datalink (CPRD). *Int. J. Epidemiol.* **44**, 827–836 (2015).
20. CPRD GOLD July 2022 dataset | CPRD. https://cprd.com/cprd-gold-july-2022-dataset.
21. Minassian, C. *et al.* Methods to generate and validate a Pregnancy Register in the UK Clinical Practice Research Datalink primary care database. *Pharmacoepidemiol. Drug Saf.* **28**, 923–933 (2019).
22. Jones, K. H., Ford, D. V., Thompson, S. & Lyons, R. A. A Profile of the SAIL Databank on the UK Secure Research Platform. *Int. J. Popul. Data Sci.* **4**, (2019).
23. Lee, S. I. *et al.* Epidemiology of pre-existing multimorbidity in pregnant women in the UK in 2018: a population-based cross-sectional study. *BMC Pregnancy Childbirth* **22**, 1–15 (2022).
24. Given, J., Casson, K., Dolk, H. & Loane, M. Evaluation of maternal medications and diseases recorded in Northern Ireland Maternity System (NIMATS) database compared to NI Enhanced Prescribing Database (EPD): a data linkage validation study. *Int. J. Popul. Data Sci.* **3**, (2018).
25. Lee, S. I. *et al.* Protocol for the development of a core outcome set for studies of pregnant women with pre-existing multimorbidity. *BMJ Open* **11**, e044919 (2021).
26. Belle Beau, A. *et al.* IMI2 821520-ConcePTION ConcePTION WP1-Moving beyond pregnancy registries to enhance our understanding of disease-related pregnancy outcomes, medication use and safety of use during pregnancy D1.2 Core evidence elements for generating medication safety evidence for pregnancy using population-based data Core data elements, design and analytical foundations.
27. Welsh Dispensing Dataset (WDDS). https://web.www.healthdatagateway.org/dataset/50ef6443-ed4b-40f9-97fb-1cfd53be6579.
28. 12 Weeks Pregnant | Pregnancy | Start for Life. https://www.nhs.uk/start4life/pregnancy/week-by-week/1st-trimester/week-12/#anchor-tabs.
29. Gail, M. H. *et al.* Design choices for observational studies of the effect of exposure on disease incidence. *BMJ Open* **9**, (2019).
30. Avillach, P. *et al.* Pilot evaluation of an automated method to decrease false-positive signals induced by co-prescriptions in spontaneous reporting databases. *Pharmacoepidemiol. Drug Saf.* **23**, 186–194 (2014).

For peer review only

The RECORD statement – checklist of items, extended from the STROBE statement, that should be reported in observational studies using routinely collected health data.

	Item No.	STROBE items	Location in manuscript where items are reported	RECORD items	Location in manuscript where items are reported
**Title and abstract**					
	1	(a) Indicate the study's design with a commonly used term in the title or the abstract (b) Provide in the abstract an informative and balanced summary of what was done and what was found		RECORD 1.1: The type of data used should be specified in the title or abstract. When possible, the name of the databases used should be included.	Multiple databases are planned to be used. This is mentioned in methods and analysis section of the abstract.
				RECORD 1.2: If applicable, the geographic region and timeframe within which the study took place should be reported in the title or abstract.	Mentioned in the methods and analysis section of the abstract.
				RECORD 1.3: If linkage between databases was conducted for the study, this should be clearly stated in the title or abstract.	No linkage involved in the study
**Introduction**					
Background rationale	2	Explain the scientific background and rationale for the investigation being reported			Introduction section, para 4
Objectives	3	State specific objectives, including any prespecified hypotheses			Aims section
**Methods**					
Study Design	4	Present key elements of study design early in the paper			Study design section within

For peer review only

					methods and analysis
Setting	5	Describe the setting, locations, and relevant dates, including periods of recruitment, exposure, follow-up, and data collection			Table 1
Participants	6	*(a) Cohort study* - Give the eligibility criteria, and the sources and methods of selection of participants. Describe methods of follow-up<br>*Case-control study* - Give the eligibility criteria, and the sources and methods of case ascertainment and control selection. Give the rationale for the choice of cases and controls<br>*Cross-sectional study* - Give the eligibility criteria, and the sources and methods of selection of participants<br><br>*(b) Cohort study* - For matched studies, give matching criteria and number of exposed and unexposed<br>*Case-control study* - For matched studies, give matching criteria and the number of controls per case		RECORD 6.1: The methods of study population selection (such as codes or algorithms used to identify subjects) should be listed in detail. If this is not possible, an explanation should be provided.<br><br>RECORD 6.2: Any validation studies of the codes or algorithms used to select the population should be referenced. If validation was conducted for this study and not published elsewhere, detailed methods and results should be provided.<br><br>RECORD 6.3: If the study involved linkage of databases, consider use of a flow diagram or other graphical display to demonstrate the data linkage process, including the number of individuals with linked data at each stage.	Table 1<br><br>Reference number 23<br><br>No linkage
Variables	7	Clearly define all outcomes, exposures, predictors, potential confounders, and effect modifiers. Give diagnostic criteria, if applicable.		RECORD 7.1: A complete list of codes and algorithms used to classify exposures, outcomes, confounders, and effect modifiers should be provided. If these cannot be reported, an explanation should be provided.	This is a signal detection study with a wide range of exposures and outcomes.
Data sources/ measurement	8	For each variable of interest, give sources of data and details			Table 2

For peer review only

		of methods of assessment (measurement). Describe comparability of assessment methods if there is more than one group			
Bias	9	Describe any efforts to address potential sources of bias			Systematic signal review section under methods and analysis
Study size	10	Explain how the study size was arrived at			Table 1
Quantitative variables	11	Explain how quantitative variables were handled in the analyses. If applicable, describe which groupings were chosen, and why			Exposure section, paragraph 2 and covariates section under methods and analysis
Statistical methods	12	(a) Describe all statistical methods, including those used to control for confounding<br><br>(b) Describe any methods used to examine subgroups and interactions<br>(c) Explain how missing data were addressed<br><br>(d) *Cohort study* - If applicable, explain how loss to follow-up was addressed<br>*Case-control study* - If applicable, explain how matching of cases and controls was addressed<br>*Cross-sectional study* - If applicable, describe analytical methods taking account of sampling strategy			(a) Statistical Analysis section, para 2 under methods and analysis<br>(b) No subgroups<br><br>(c) Covariates section under methods and analysis<br>(d) No matching

For peer review only

		(e) Describe any sensitivity analyses			(e) No sensitivity analyses
Data access and cleaning methods		..		RECORD 12.1: Authors should describe the extent to which the investigators had access to the database population used to create the study population.<br><br>RECORD 12.2: Authors should provide information on the data cleaning methods used in the study.	Table 1<br><br>Described elsewhere (Reference 23)
Linkage		..		RECORD 12.3: State whether the study included person-level, institutional-level, or other data linkage across two or more databases. The methods of linkage and methods of linkage quality evaluation should be provided.	No linkage
**Results**					
Participants	13	(a) Report the numbers of individuals at each stage of the study (*e.g.*, numbers potentially eligible, examined for eligibility, confirmed eligible, included in the study, completing follow-up, and analysed)<br>(b) Give reasons for non-participation at each stage.<br>(c) Consider use of a flow diagram		RECORD 13.1: Describe in detail the selection of the persons included in the study (*i.e.,* study population selection) including filtering based on data quality, data availability and linkage. The selection of included persons can be described in the text and/or by means of the study flow diagram.	Study population section under methods and analysis
Descriptive data	14	(a) Give characteristics of study participants (*e.g.*, demographic, clinical, social) and information on exposures and potential confounders<br>(b) Indicate the number of participants with missing data for each variable of interest			This is a protocol paper

For peer review only

		(c) *Cohort study* - summarise follow-up time (*e.g.*, average and total amount)			
Outcome data	15	*Cohort study* - Report numbers of outcome events or summary measures over time<br>*Case-control study* - Report numbers in each exposure category, or summary measures of exposure<br>*Cross-sectional study* - Report numbers of outcome events or summary measures			This is a protocol paper
Main results	16	(a) Give unadjusted estimates and, if applicable, confounder-adjusted estimates and their precision (e.g., 95% confidence interval). Make clear which confounders were adjusted for and why they were included<br>(b) Report category boundaries when continuous variables were categorized<br>(c) If relevant, consider translating estimates of relative risk into absolute risk for a meaningful time period			This is a protocol paper
Other analyses	17	Report other analyses done—e.g., analyses of subgroups and interactions, and sensitivity analyses			This is a protocol paper
**Discussion**					
Key results	18	Summarise key results with reference to study objectives			This is a protocol paper
Limitations	19	Discuss limitations of the study, taking into account sources of potential bias or imprecision.		RECORD 19.1: Discuss the implications of using data that were not created or collected to answer the specific research question(s). Include	Systematic Signal review section under methods and analysis

For peer review only

		Discuss both direction and magnitude of any potential bias		discussion of misclassification bias, unmeasured confounding, missing data, and changing eligibility over time, as they pertain to the study being reported.	
Interpretation	20	Give a cautious overall interpretation of results considering objectives, limitations, multiplicity of analyses, results from similar studies, and other relevant evidence			This is a protocol paper
Generalisability	21	Discuss the generalisability (external validity) of the study results			This is a protocol paper
**Other Information**					
Funding	22	Give the source of funding and the role of the funders for the present study and, if applicable, for the original study on which the present article is based			Funding section
Accessibility of protocol, raw data, and programming code		..		RECORD 22.1: Authors should provide information on how to access any supplemental information such as the study protocol, raw data, or programming code.	This is a protocol paper

*Reference: Benchimol EI, Smeeth L, Guttmann A, Harron K, Moher D, Petersen I, Sørensen HT, von Elm E, Langan SM, the RECORD Working Committee. The REporting of studies Conducted using Observational Routinely-collected health Data (RECORD) Statement. *PLoS Medicine* 2015; in press.

*Checklist is protected under Creative Commons Attribution (CC BY) license.

BMJ Open

Detection and evaluation of signals associated with exposure to individual and combination of medications in pregnancy: A signal detection study protocol

Journal:	*BMJ Open*
Manuscript ID	bmjopen-2023-073162.R1
Article Type:	Protocol
Date Submitted by the Author:	20-Jul-2023

Complete List of Authors:	Subramanian, Anuradhaa; University of Birmingham, Institute of Applied Health Research<br>Lee, Siang Ing; University of Birmingham Institute of Applied Health Research<br>Hemali Sudasinghe, Sudasing Pathirannehelage Buddhika; University of Birmingham, Institute of Applied Health Research<br>Wambua, Steven; University of Birmingham, Applied health research<br>Phillips, Katherine; University of Birmingham, Applied health research<br>Singh, Megha; University of Birmingham, Applied health research<br>Azcoaga-Lorenzo, Amaya; University of Saint Andrews School of Medicine, Division of Population and Behavioural Sciences; Instituto de Investigación Sanitaria Fundación Jimenez Diaz, Hospital Rey Juan Carlos. Research Network on Chronicity, Primary Care and Health Promotion-RICAPPS (RICORS)<br>Cockburn, Neil; University of Birmingham, Applied health research<br>Wang, Jingya; Institute of Applied Health Research University of Birmingham<br>Fagbamigbe, Adeniyi; University of St Andrews, PBS; University of Ibadan, EMS<br>Usman, Muhammad; University of Ibadan, University of St Andrews<br>Damase-Michel, Christine; Service de Pharmacologie Médicale, CHU Toulouse, Université Toulouse III<br>Yau, Christopher; The University of Manchester, Division of Informatics, Imaging and Data Sciences, Faculty of Biology Medicine and Health<br>Kent, Lisa; Queen's University Belfast, Centre for Public Health<br>McCowan, Colin ; University of St. Andrews<br>OReilly, Dermot; Queens University Belfast, Epidemiology and Public Health<br>Santorelli, Gillian; Bradford Teaching Hospitals NHS Foundation Trust<br>Hope, Holly; Manchester University<br>Kennedy, Jonathan; Swansea University, College of Medicine<br>Mhereeg, Mohamed; Swansea University Medical School<br>Abel, Kathryn ; Institute of Brain Behaviour and Mental Health, Medical and Human Sciences; Manchester Mental Health & Social Care Trust<br>Eastwood, Kelly-Ann; University Hospitals Bristol and Weston NHS Foundation Trust<br>Black, Mairead; University of Aberdeen<br>Loane , Maria ; University of Ulster<br>Moss, Ngawai; Queen Mary University of London

	Brophy, Sinead; Swansea University, College of Medicine<br>Brocklehurst, Peter; University of Birmingham<br>Dolk, Helen; Ulster University - Jordanstown Campus, Institute for Nursing and Health Research<br>Nelson-Piercy, Catherine; Guy’s & St Thomas’ Foundation Trust<br>Nirantharakumar, Krishnarajah; University of Birmingham
**Primary Subject Heading**:	Reproductive medicine
Secondary Subject Heading:	Epidemiology, Obstetrics and gynaecology, Public health
Keywords:	OBSTETRICS, Maternal medicine < OBSTETRICS, EPIDEMIOLOGY

SCHOLARONE™
Manuscripts

Detection and evaluation of signals associated with exposure to individual and combination of medications in pregnancy: A signal detection study protocol

For peer review only

Authors: Anuradhaa Subramanian[1], Siang Ing Lee[1], Sudasing Pathirannehelage Buddhika Hemali Sudasinghe[1], Steven Wambua[1], Katherine Phillips[1], Megha Singh[1], Amaya Azcoaga-Lorenzo[2,3], Neil Cockburn[1], Jingya Wang[1], Adeniyi Francis Fagbamigbe[2,4], Muhammad Usman[3], Christine Damase-Michel[5,6], Christopher Yau[7,8], Lisa Kent[9], Colin McCowan[2], Dermot O'Reilly[9], Gillian Santorelli[10], Holly Hope[11], Jonathan I Kennedy[12], Mohamed Mhereeg[12], Kathryn M Abel[11,13], Kelly-Ann Eastwood[9, 14], Mairead Black[15], Maria Loane[16], Ngawai Moss[17], Sinead Brophy[12], Peter Brocklehurst[1#], Helen Dolk[18#], Catherine Nelson-Piercy[19#], Krishnarajah Nirantharakumar[1#], on behalf of the MuM-PreDiCT Group.

[#] joint senior authors

Corresponding author: Prof Krishnarajah Nirantharakumar
k.nirantharan@bham.ac.uk

Affiliation:

1. Institute of Applied Health Research, University of Birmingham, Birmingham, UK
2. Division of Population and Behavioural Sciences, School of Medicine, University of St Andrews, UK
3. Hospital Rey Juan Carlos, Instituto Investigacion Sanitaria Fundacion Jimenez Diaz
4. Department of Epidemiology and Medical Statistics, College of Medicine, University of Ibadan, Nigeria
5. Medical and Clinical Pharmacology, School of Medicine, Université Toulouse III, France
6. INSERM, Center for Epidemiology and Research in Population Health (CERPOP), France
7. Division of Informatics, Imaging and Data Sciences, Faculty of Biology Medicine and Health, The University of Manchester, UK
8. Health Data Research UK
9. Centre for Public Health, Queen's University of Belfast, UK
10. Bradford Institute for Health Research, UK
11. Centre for Women's Mental Health, Division of Psychology and Mental Health, School of Health Sciences, Faculty of Biology Medicine & Health, The University of Manchester, UK
12. Data Science, Medical School, Swansea University, UK
13. Greater Manchester Mental Health NHS Foundation Trust, Manchester, UK
14. St Michael's Hospital, University Hospitals Bristol NHS Foundation Trust, UK
15. Aberdeen Centre for Women's Health Research, School of Medicine, Medical Science and Nutrition, University of Aberdeen, UK
16. The Institute of Nursing and Health Research, Ulster University, UK
17. Patient and public representative, London, UK
18. School of Medicine, Ulster University, UK.
19. Guy's and St. Thomas' NHS Foundation Trust, UK

Abstract

Introduction

Considering the high prevalence of polypharmacy in pregnant women and the knowledge gap in the risk-benefit safety profile of their often-complex treatment plan, more research is needed to optimise prescribing. In this study, we aim to detect adverse and protective effect signals of exposure to individual and pairwise combinations of medications during pregnancy.

Methods and analysis

Using a range of real-world data sources from the United Kingdom (UK), we aim to conduct a pharmacovigilance study to assess the safety of medications prescribed during the preconception period (three months prior to conception) and first trimester of pregnancy. Women aged between 15-49 years with a record of pregnancy within the Clinical Practice Research Datalink (CPRD) Pregnancy Register, the Welsh Secure Anonymised Information Linkage (SAIL), the Scottish Morbidity Record datasets (SMR), and the Northern Ireland Maternity System (NIMATS) will be included.

A series of case control studies will be conducted to estimate measures of disproportionality, detecting signals of association between a range of pregnancy outcomes and exposure to individual and combinations of medications.

A multidisciplinary expert team will be invited to a signal detection workshop. By employing a structured framework, signals will be transparently assessed by each member of the team using a questionnaire appraising the signals on aspects of temporality, selection, time, and measurement related biases and confounding by underlying disease or co-medications. Through group discussion, the expert team will reach consensus on each of the medication exposure-outcome signal, thereby excluding spurious signals, leaving signals suggestive of causal associations for further evaluation.

Ethics and dissemination

Ethical approval has been obtained from the Independent Scientific Advisory Committee, SAIL Information Governance Review Panel, University of St. Andrews Teaching and Research Ethics Committee and Office for Research Ethics Committees Northern Ireland (ORECNI) for access and use of CPRD, SAIL, SMR and NIMATS data respectively.

Strengths and limitations of this study

- A comprehensive range of medication exposures prescribed both individually and as a combination with another medication within primary care is included in this study, providing a wide opportunity to detect and evaluate signals of adverse and protective effect during pregnancy
- This study utilises a wide range of data sources obtaining prescription data from primary care (from Clinical Practice Research Datalink and Secure Anonymized Information Linkage), and the community (from Prescribing Information System). However, the study is limited by the unavailability of prescription data from secondary care
- The results of this signal detection study are limited by the quality of routinely collected exposure and outcome data used for this study
- This signal detection study is susceptible to type 1 and type 2 errors owing to limitations in terms of multiple testing and insufficient sample size respectively
- While the results of this exploratory signal detection study may provide useful signals, they may suffer from biases related to confounding, and must be followed up by methodologically rigorous pharmacoepidemiological studies focussed on each signal separately

For peer review only

Introduction

Conventional methods of drug development from discovery to pre-clinical and clinical research and review are expensive, time consuming and disproportionately concentrate on disease areas that predominantly affect men.(1) The effect of this can be seen in the female dominance of adverse drug effect reporting, especially during the period of reproductive age.(2) Pregnant and breast-feeding women are further inclined to become `therapeutic orphans', by their routine exclusion from clinical trials, given the struggle to insure such trials involving pregnant women. Pregnant women are further disadvantaged by having to arduously navigate through multifarious social influences, some losing their autonomy in medical decision making around taking medications or in their choice to participate in a clinical trial during pregnancy.(3,4)

In light of the urgent action required to develop safe medications for use during pregnancy, the 'Healthy Mum, Healthy Baby, Healthy Future' report by the Birmingham Health Partners advocates inclusion of pregnant women in clinical trials unless there are specific safety concerns.(5) The report also makes a case to pilot a 'Maternal Investigation Plan' similar to the 'Paediatric Investigation Plan' implemented by European Union, to make licensing compulsory for use of medications during pregnancy.

In the absence of safety signals for medication exposure during pregnancy from clinical trial data, information on teratogenicity is limited, and is only prospectively collected from national surveillance data, available from resources such as UK Teratology Information Service (UKTIS).(6) This limited availability of robust evidence on medication use during pregnancy has led to women often being prescribed medications 'off-label' in practice.(7) Without appropriate medication safety information, the decision to continue, discontinue or switch their medication falls to the women themselves and their healthcare providers.(8)

We have previously found that the prevalence of polypharmacy in the United Kingdom (UK), during the first trimester of pregnancy alone, has increased from 8.7% to 18.7% over the last two decades.(9) While polypharmacy itself may be essential and beneficial in the management of multiple chronic conditions in combination with pregnancy related complications, inappropriate polypharmacy may cause preventable adverse drug events (ADEs) due to drug-drug interactions.(10) Considering the high prevalence of polypharmacy among women of reproductive age and the knowledge gap in safety profiles of complex treatment plan,(9,11–13) more research is urgently needed to optimise prescribing by detecting signals of adverse outcomes following exposure to combination of medications.

Real world data (RWD) can be used to assess the post-market safety of the use of individual and combinations of medication during pregnancy, generating evidence to support regulatory decision making. In addition, RWD also provides an opportunity for repurposing of approved medications as prophylactic treatments for pregnancy related complications.(14) Real world data-based signal detection involves a systematic step-by-step approach(15–17): (1) identifying real world data source and inspecting its feasibility in accurately ascertaining exposures and outcomes to support identification of safety signals, (2) listing the outcomes (adverse events) and exposures (medications) of interest, (3) generating measures of statistical association for large sets of exposure-outcome pairs with methodological design to limit confounding, (4) reviewing identified signals by a multidisciplinary team and considering sources of bias that lead to false positive signals to ensure contextual interpretation and (5)

For peer review only

strengthening and confirming screened signals using rigorous pharmaco-epidemiological studies or clinical trials.

The feasibility of mining real world data to detect adverse and protective effect signals of individual medications during pregnancy has already been established.(16,18) In this study, as part of the "Multimorbidity and Pregnancy: Determinants, Clusters, Consequences and Trajectories (MuM-PreDiCT)" consortium, we aim to develop and implement a systematic signal detection methodology to determine adverse and protective signals from both individual and combinatorial medication exposure on the incidence of various pregnancy outcomes, using a set of large primary care, secondary care and maternity care databases within the UK.

Aims

The study aims to evaluate the feasibility of conducting a signal detection study estimating and evaluating the adverse and protective signals of medications prescribed during the preconception period and the first trimester of pregnancy on the incidence of various pregnancy outcomes using real world data

The key objectives include:

(1) To scope real world data sources in UK, and to check their feasibility for signal detection by exploring the range of medications prescribed and recorded within said data sources during the preconception period and first trimester of pregnancy
(2) To identify a core list of pregnancy outcomes, and to explore the feasibility of capturing these outcomes within said data sources
(3) To apply suitably developed methods from published literature to estimate measures of disproportionality for each of the exposure-outcome pairs and detect signals
(4) To systematically review the detected signals through a multidisciplinary expert committee workshop, using a pre-specified structured questionnaire exploring sources of false positives.

For peer review only

Methods and analysis

Data source

Four population-based data sources spanning all four nations of the UK will provide data for this signal detection study. These include primary care data sources such as Clinical Practice Research Datalink (CPRD, from all four nations) and The Secure Anonymized Information Linkage (SAIL, Wales) and secondary care data sources such as Hospital Episode Statistics (HES, England), Scottish Morbidity Records (SMR) with linked community prescription data (Scotland), and Northern Ireland Maternity System (NIMATS) with linked Enhanced Prescribing Database (EPD, Northern Ireland). The data sources are scoped, and their feasibility for this signal detection study is assessed and tabulated in Table 1-4.

(1) Clinical Practice Research Datalink and Hospital Episode Statistics

CPRD Gold and Aurum contains anonymized, longitudinal medical records of over 20 and 39 million patients in the UK collected by over 985 and 1,489 participating general practices

respectively as part of their care and support.(19) It currently covers general practices that use the Vision and EMIS software, and collects data from 20% of general practices in the UK.(20) It includes data on demographics, diagnoses and prescriptions. Linkage to area-based deprivation index known as the Index of Multiple Deprivation (IMD) and Hospital Episodes Statistics (HES) is available for 75% of patients in England, whose general practices have consented to the CPRD linkage scheme.

CPRD Gold contains data on medications prescribed within primary care with associated prescription time stamps, encoded using drug codes that are assigned and categorized according to British National Formulary (BNF) item codes. However, CPRD Gold may be limited by their unavailability of secondary care and over the counter medication data, and data on whether these prescriptions were dispensed.

Within CPRD, the CPRD Pregnancy Register is an algorithm that takes information from maternity, antenatal and birth health records from primary care to detect pregnancies and their outcomes.(21) A subset of the Pregnancy Register, Mother Baby Linked (MBL) data further allows for studying outcomes in the children.

(2) The Secure Anonymized Information Linkage

The SAIL databank, a population level database in Wales, is a repository of anonymized health and socio-economic administrative data that provide linkage at an individual level.(22) It holds health data for 4.8 million people and includes data contributed by 80% of Welsh general practices. National Community Child Health Dataset, GP records and hospital records have been used to detect pregnancies in SAIL.(23) In addition, patient level linkage to the Welsh Longitudinal General Practice dataset and the Welsh Demographic Service dataset has been used to obtain data on diagnoses, prescriptions and demographics data respectively. The Welsh Dispensing Data Set (WDDS) containing information on general practitioner (GP) prescribed medications and dispensed medications by community contractors has been linked to the SAIL databank. Similar to CPRD, SAIL may be limited by its unavailability of secondary care and over the counter medication data and data on whether the prescriptions were dispensed.

(3) Scottish Maternity Records and linked data sources

The Scottish Maternity Records (SMR02) will be linked to data from Hospital Admissions (SMR01), Mental Health Inpatients (SMR04), Accident and Emergency and the Demography and Death registries,(23) covering diagnoses and demographic data for all inpatient stays and day cases for residents in Scotland. Maternity records (SMR02) or pregnancy-related hospital admissions (SMR01) allows for identification of pregnancies, and prescription data of medications dispensed in the community can be obtained from linked Prescribing Information System (PIS).

(4) Northern Ireland Maternity System

NIMATs holds demographic and clinical information on mothers and infants. It captures data relating to the current complete maternity process, but also contains details about the mother’s past medical and obstetric history. NIMATs contains information on medications that mothers self-reported to have taken during pregnancy. In addition to self-reported data on

For peer review only

medications, linkage of NIMATs to EPD allows for analysis of prescription data issued by GPs.(24)

Study design

A series of case control studies will be conducted to estimate measures of disproportionality, detecting signals of association between each of the pregnancy outcomes of interest and exposure to individual or combination of medications prescribed during the preconception period and first trimester of pregnancy.

Study population

For this feasibility study, we aim to conduct the analyses separately across all four databases described in the section above. Women aged between 15-49 years with a pregnancy recorded within the CPRD Gold Pregnancy Register (all four nations), National Community Child Health data (Wales), SMR02 (Scotland), and NIMATS (Northern Ireland), within the study period customized to the period of data availability within each data source will be eligible for inclusion in this study (Table 1-4). Data standard quality checks for each of the databases, and eligibility criteria for inclusion is presented in a previous publication.(23) Pregnancy start dates will be either derived using a pre-defined algorithm or used as reported within the said data source (Table 1-4), and will be used to define exposure and outcome time windows.

Outcomes

The MuM-PreDiCT and ConcePTION consortium have developed core outcome sets (a minimum set of recommended maternal and offspring outcomes) for studies of pregnant women with multiple long-term conditions and for studies generating medication safety evidence respectively.(25,26) The outcome set was reviewed by a study advisory group panel comprising of general practitioners, obstetricians, obstetric physicians and experienced users of the available data sources, to identify the pregnancy outcomes' suitability and feasibility for inclusion in this signal detection study. The availability, prevalence, quality, and completeness of data recording of these outcomes within the said data sources were used as criteria to determine the feasibility of the outcome of interest. Outcomes with poor recording in a specific database may not be included in the analysis using that database to avoid noisy signals due to insufficient power or case misclassification. Furthermore, outcomes that cannot be considered as a medication adverse event such as termination of pregnancy, outcomes that were too broad or non-specific such as involvement of patients in care decisions, and neonatal outcomes that were too narrow and reflective of prematurity such as intubation/ventilation requirement were excluded. The final list of outcomes to be considered for inclusion in this study is available in Table 5, and the operational definitions of these outcomes is available in Supplementary Table 1.

Exposure

All medications prescribed within primary care [as in the CPRD Gold database (all four nations) and EPD (Northern Ireland)] and all medications dispensed in the community [as in the PIS database (Scotland)] have a BNF code associated with them in the UK. Dispensed medications in WDDS (Wales) are coded based on the Dictionary of Medicines and Devices (DM+D). Using a complete extract of the dictionary,(27) each dispensed item within the dictionary can be mapped to a corresponding BNF code.

For peer review only

Analysis stratified by BNF codes has been established in a previous analysis, wherein using a dictionary of medications prescribed within primary care , we stratified the medications prescribed according to their 4-digit BNF code (BNF chapter, section, paragraph, and sub-paragraph), screened and selected 577 BNF items that were pharmacological agents with therapeutic action.(9)

Using a similar strategy, we will ascertain the exposure information for a range of medications stratified by their BNF code specifically within four crucial time windows: (1) preconception period (up to 90 days prior to the start of pregnancy) (2) first trimester of pregnancy (first 12 weeks of pregnancy), (3) second trimester of pregnancy (between 13 and 26 weeks of pregnancy), and (4) third trimester of pregnancy (between 27 weeks and end of pregnancy) . (28) However, the exposure ascertainment within these windows will be restricted further to the time prior to outcome diagnosis to preserve exposure-outcome temporality. For outcomes that occur during the first trimester of pregnancy such as miscarriage, the exposure time window will be restricted to the preconception period and first trimester only. Furthermore, we will ascertain the exposure information for a range of medication pairs that are prescribed concurrently within the same exposure window to assess adverse and protective effect signals associated with medications prescribed in pairs.

While the data sources comprehensively capture medicines prescribed within primary care and within the community, the study is limited to medicines marketed and prescribed in the UK. Furthermore, signals from over the counter medications cannot be captured within this study.

Covariates

The following demographic and health characteristics will be obtained from the four datasets; maternal age at the start of pregnancy, ethnicity, smoking status as recorded prior to the start of pregnancy, pre-gravid body mass index (BMI) and a wide range of comorbidities. Patients with missing data on smoking status, pre-gravid BMI, and ethnicity will be categorized into a separate missing category within the corresponding variable. The list of 79 pre-existing long-term comorbidities for which baseline data were extracted, and their definitions is presented in a previous publication. (23) Ethnicity will be categorized as White, South Asian, Black Afro-Caribbean, mixed ethnic background, and other ethnic minority groups including Chinese. Latest BMI recorded prior to the start of pregnancy will be considered as pre-gravid BMI, and will be categorized according to the World Health Organization definition as underweight (<18.5 kg/m^2), healthy weight (18.5-24.9 kg/m^2), overweight (25-29.9 kg/m^2), and obese (≥30 kg/m^2).(29) Smoking status of patients prior to the start of pregnancy will categorise patients as current smokers, ex-smokers, and never-smokers.

Statistical analysis

Description of baseline characteristics

Patient covariates will be summarized and stratified by their outcome status using numbers and percentages for categorical variables and mean (standard deviation) or median (interquartile range) for continuous variables.

Statistical analysis

Odds ratio (OR) will be considered as the primary measure of disproportionality, and will be estimated for each of the exposure-outcome pairs using a series of univariate logistic

For peer review only

regression models. In a series of adjusted logistic regression models, the exposure-outcome relationships will be adjusted for covariates including age at start of pregnancy, pre-gravid BMI, ethnicity, smoking status, and a disease risk score (DRS) to obtain adjusted odds ratios with 95% confidence intervals. DRS will be generated for each of the outcomes using logistic regression models considering the outcome as a dependent variable and a range of pre-existing long-term conditions as independent variables. This is done to limit the effect of confounding attributable to prescriptions issued in order to manage the underlying long-term conditions. Relative decrease in p-value (p-RD) prior to and after adjustment for covariates will be calculated using the formula below.(16)

$$p - RD = \frac{p\ (before\ adj\ for\ DRS) - p(after\ adj\ for\ DRS)}{p\ (before\ adjustment)}$$

In addition to statistical measures of disproportionality, descriptive data stratified by their outcome status will be presented. These include numbers and proportions of eligible pregnancies with a prescription of the individual medication or medication combinations (pairs) during the two separate exposure windows.

Benjamini–Hochberg correction for multiple testing will be applied with a threshold of 0.20.

The analyses in each of the four datasets will be conducted separately. Signals arising from each of the four data sources will be reviewed side-by-side through a systematic signal review workshop described below.

Systematic Signal review

A multidisciplinary expert team comprising of epidemiologists, general practitioners, obstetricians, obstetric physicians, pharmacists, data scientists, experts in genetics, internal medicine and pharmacovigilance, and researchers with expertise on the outcome of interest will be invited to a signal detection workshop. The workshop is aimed at collaboratively working and screening potential signals to identify and exclude signals that are likely to be affected by bias and confounding, leaving signals suggestive of causal associations to be further evaluated in future studies.

A list of all the exposure-outcome pairs, the adjusted and unadjusted OR along with 95% confidence intervals, and p-RD will be presented to the multidisciplinary team in the order of statistical significance and strength of association. Exposure-outcome pairs with a clinically significant strength of association without statistical significance will also be provided to the review team to avoid false negatives.

A checklist with the following items will be provided to the review team: (1) confirmation of exposure – outcome temporality to limit the possibility of reverse causality,(30) (2) consideration of concomitant medications to limit the possibility of co-prescription bias,(31) (3) consideration medical history, (4) consideration of demographic features, and (5) consideration of underlying disease and other alternative explanations to limit the possibility of confounding. After consideration of the checklist items, we will request each reviewer to mark the exposure-outcome pairs with three possible response options: (1) "already established", (2) "warrant further investigation" and, (3) "dismissed", with reasons for each response. In a group discussion, conflicting responses will be discussed, and a consensus will be made.

For peer review only - http://bmjopen.bmj.com/site/about/guidelines.xhtml

Ethics approval and consent to participate

CPRD has ethics approval from the Health Research Authority to support research using anonymised patient data. Use of CPRD and linked HES data for this study is approved by the Independent Scientific Advisory Committee. Use of SAIL databank for this study is approved by the SAIL Information Governance Review Panel. Use of SMR data for this study is approved by the School of Medicine Ethics Committee, acting on behalf of the University of St. Andrews Teaching and Research Ethics Committee. SAIL and SMR data will be analysed within a Safe Haven Research environment. Use of NIMATS and EPS data for this study is approved by the Office for Research Ethics Committees Northern Ireland (ORECNI) and the Honest Broker Governance Board.

Consent for publication

As the study data will be de-identified, consent for publication is not required.

Competing interests

None declared.

Funding statement

This work is independent research funded by the Strategic Priority Fund "Tackling multimorbidity at scale" programme (grant number MR/W014432/1) delivered by the Medical Research Council and the National Institute for Health Research in partnership with the Economic and Social Research Council and in collaboration with the Engineering and Physical Sciences Research Council. The views expressed are those of the author and not necessarily those of the funders, the NIHR or the UK Department of Health and Social Care. The funders had no role in study design, data collection and analysis, decision to publish, or preparation of the manuscript.

Patient and Public Involvement

Patient and public involvement (PPI) have been extensive from design to dissemination of research outputs from the MuM-PreDiCT group. Our PPI representatives have provided advice on the importance and relevance of this study, and helped shape the research question. PPI representatives were also involved in screening and identifying the pregnancy outcomes of interest that are relevant to this study. One of the PPI representatives (NM) also co-authored this protocol. PPI representatives will be involved in the interpretation of the results in the future.

Contributorship statement

Our authors list includes PPI co-investigator NM. AS, SIL, KP, AA-L, CD-M, CM, DOR, HH, JIK, KMA, K-AE, MB, ML, NM, SB, PB, HD, CNP and KN conceived the study and contributed to the study design. AS led the development of the protocol and drafted the initial manuscript with contribution from SIL, SW, KP, JW, LK and JIK, and supervision from AA-L, CD-M, CM, DOR, KMA, MB, ML, SB, PB, HD, CNP and KN. All authors critically

For peer review only

reviewed and revised the protocol drafts, and agreed on the final draft manuscript for submission.

For peer review only

Table 1: Scoping real world data source from England to check its' feasibility for signal detection

Data	Features of the dataset	
CPRD	**Setting**	Primary care practices using the Vision® software system
	**Geographical region**	All four nations of the UK, with relatively few practices heavily concentrated in three conurbations and the South
	**Coverage**	4% of the UK
	**Linkage availability to**	• Pregnancy Register and Mother-Baby Linked data<br>• Deprivation data (Patient postcode linked deprivation measures, Practice postcode linked deprivation measures, Index of Multiple Deprivation (IMD), Townsend Deprivation Index, Carstairs Index, Rural-Urban Classification)<br>• Office of National Statistics (ONS) Death Registration Data<br>• Hospital Episode Statistics (HES)<br>• Cancer Registration Data (However, this is not accessed for this study)<br>• Congenital anomaly register (However, this is not accessed for this study)
	**Data content (with linkage)**	• Patient demographics<br>• Consultation details<br>• Symptoms, signs and diagnoses<br>• Referrals to external care<br>• Immunisation details<br>• Records of test data in the GP system<br>• All prescriptions (medications and appliances) issued by the GP
	**Period of data availability**	January 2000 to July 2022
	**Feasibility to identify pregnancy episodes?**	Yes, using CPRD Pregnancy Register

For peer review only

	**Sample size – number of pregnancy episodes**	1.5 million pregnancies
	**Definition of pregnancy start date**	As derived within the CPRD Pregnancy Register
	**Feasibility to identify exposures?**	Yes, using prescription records on the GP system. This is coded using the Dictionary of Medicines and Devices (DM+D). Each product within the dictionary is associated with a BNF code to which the product belongs. The range of exposures for this study will be defined using the BNF code.<br><br>Limitation: The data relates to primary care prescribing only. Secondary care prescribing data, over the counter medications, and data on adherence to treatments are unavailable using this data source. Furthermore, absence of dispensing data may cause exposure misclassification in the instance of prescriptions that are issued but not dispensed
	**Feasibility to identify pregnancy outcomes?**	Yes, using Read code diagnoses within primary care or ICD-10 diagnoses within linked HES data.

For peer review only

Table 2: Scoping real world data source from Wales to check its' feasibility for signal detection

Data	Features of the dataset	
SAIL	Setting	A population level database in Wales. SAIL is a repository of anonymized health and socio-economic administrative data that provides linkage at an individual level using Trusted Third Party (TTP) in the NHS Wales Informatics Service (NWIS).
	Geographical region	Wales
	Coverage	The database contains 90% of all the GP data in Wales and 100% of all hospital admissions (PEDW)
	Linkage availability to	• Primary care data (from Wales Longitudinal General Practice (WLGP))<br>• Secondary care data (from inpatient hospital admissions, inpatient from Patient Episode Database for Wales (PEDW) and outpatient from Outpatient Database for Wales (OPDW)<br>• The National Community Child Health (NCCH)<br>• The Maternal Indicators (MIDS)<br>• The Welsh Dispensing Data Set (WDDS)<br>• The Welsh Demographic Service Dataset (WDSD)<br>• Office of National Statistics (ONS) Annual District Birth Extract<br>• Office of National Statistics (ONS) Annual District Death Extract<br>• Congenital Anomaly Register and Information Service (CARIS)
	Data content (with linkage)	• (WLGP): Signs, symptoms, test results, diagnoses, prescribed treatment, referrals for specialist treatment<br>• (PEDW): attendance and clinical information, diagnoses and operations performed. (OPDW): attendance information for all NHS Wales hospital outpatient appointments<br>• (NCCHD): Comprises information pertaining to birth registration, monitoring of child health examinations, and immunisations.<br>• (MIDS): contains data relating to the woman at initial assessment and to the mother and baby (or babies) for all births.

For peer review only

		• (WDDS): containing information on GP prescribed medications, dispensed medications by community contractors have been linked to the SAIL databank.<br>• (WDSD): used to extract Lower-layer Super Output Area (LSOA) version 2011 information associated with area-level deprivation (the Welsh Index for Multiple Deprivation (WIMD) version 2019)<br>• All births in Wales collected from birth registrations.<br>• All deaths relating to Welsh residents, including those that died out of Wales<br>Information about foetuses or babies who has or is suspected of having a congenital anomaly,
	**Period of data availability**	2000-2022
	**Feasibility to identify pregnancy episodes?**	Yes, using GP, PEDW, NCCHD, and MIDS datasets
	**Sample size – number of pregnancy episodes**	~475,000 pregnancies (from the year 2000 onwards)
	**Definition of pregnancy start date**	Pregnant women can be identified as any woman who have pregnancy codes in the WLGP data, in hospital admissions (PEDW) for pregnancy, or mothers in the NCCH or MIDS data with the baby birth date (Pregnancy end date) and gestational age at birth available.
	**Feasibility to identify exposures?**	Yes, data covers prescriptions that are prescribed in Wales by GP's (general medical practitioners) and non-medical prescribers that have prescribed on behalf of the GP practice, that are then dispensed in the community within Wales. The data includes all prescribed medicines, dressings and appliances that are dispensed each month. Information includes - the number of prescription items dispensed by each community pharmacy in Wales, broken down by the GP practice in which they are prescribed, and also the number of prescription items prescribed in each GP practice in Wales broken down by the pharmacy that dispensed those items.<br><br>Limitation - The data relates to primary care prescribing only. Secondary care prescribing data, over the counter medications, and data on adherence to treatments are unavailable using this data source
	**Feasibility to identify pregnancy outcomes?**	Yes, using Read code diagnoses within primary care or ICD-10 diagnoses within hospital admissions data

For peer review only

Table 3: Scoping real world data source from Scotland to check its' feasibility for signal detection

Data	Features of the dataset	
SMR	**Setting**	Scottish Morbidity Records (SMR) from secondary care data
	**Geographical region**	Scotland
	**Coverage**	All obstetric inpatients and day cases from maternity hospitals in Scotland
	**Linkage availability to**	• Community Prescriptions
	**Data content (with linkage)**	• SMR00 - Outpatients<br>• SMR01 – Inpatient and day cases<br>• SMR02 - Maternity<br>• SMR04 – Mental Health<br>• SMR06 – Cancer<br>• Accident & Emergency<br>• NRS Deaths<br>• Prescribing Information System (community)<br>• Scottish Birth Record (SBR)<br>• National Records of Scotland (NRS) Infant Deaths<br>• NRS Stillbirths<br>• Scottish Linked Congenital Condition Dataset (SLiCCD) (However, this is not accessed for this study)
	**Period of data availability**	SMR02 (pregnancy identification) from 2008-2021 with historical data from women<br>SMR01, SMR00, PIS and A&E database for the first 5 years of life on the children
	**Feasibility to identify pregnancy episodes?**	Yes
	**Sample size – number of pregnancy episodes**	670, 811 births + pregnancies not ending in births
	**Definition of pregnancy start date**	Based on gestational age at booking appointment and or outcome of pregnancy

For peer review only

	**Feasibility to identify exposures?**	Yes, using prescription from the community. This is coded using the Dictionary of Medicines and Devices (DM+D). Each product within the dictionary is associated with a BNF code to which the product belongs. The range of exposures for this study will be defined using the BNF code.<br><br>Limitation: The data relates to primary care prescribing only. Secondary care prescribing data, over the counter medications, and data on adherence to treatments are unavailable using this data source
	**Feasibility to identify pregnancy outcomes?**	Only secondary care

For peer review only

Table 4: Scoping real world data source from Northern Ireland to check its' feasibility for signal detection

Data	Features of the dataset	
NIMATS	Setting	NIMATs is available in all five Health Trust areas across Northern Ireland (NI), within each hospital providing maternity services (11 hospitals in total). Access to NIMATS is also available to midwives/clerical staff in various community clinics across NI to allow for booking appointments to be recorded.
	Geographical region	Northern Ireland
	Coverage	100% within Northern Ireland; full coverage from 2011
	Linkage availability to	• NHAIS (Health Register) –<br>○ GP Patient Registration Index<br>○ Household characteristics (e.g. single person households)<br>○ Linkage to GRO (– Maternal deaths; Infant deaths (may be under-represented due to inability to link some infants to health records via Health & Care Number));<br>○ Via postcode,<br>○ NI Multiple Deprivation Measure (NIMDM) – area level deprivation, usually supplied as quintile or decile<br>○ Settlement Band Classification for urban/rural classification (2005-2014) (Post-code used only by HBS staff to aid linkage; not available to researchers)<br>○ Land & Property Services – rateable value of property (another measure of deprivation)<br>• Enhanced Prescribing Database (EPD) - database includes information on all medications that have been prescribed by General Practitioners and dispensed to patients in NI.<br>• Secondary care data – includes hospital episodes from The Patient Administration System (PAS) and ED attendance data.<br>• NI Cerebral Palsy Register

For peer review only

	**Data content (with linkage)**	• Demographic information<br>• Clinical information on mothers, pregnancies and infants – delivery, investigations, medications, hospital stays, postnatal complications<br>• Mother's past medical and obstetric history.
	**Period of data availability**	2011-2022
	**Feasibility to identify pregnancy episodes?**	Yes. the mother is identified by her unique Health & Care Number, but she has a different hospital maternity number for each pregnancy.
	**Sample size – number of pregnancy episodes**	~300,000 pregnancies
	**Definition of pregnancy start date**	Can be derived from NIMATS variable EDC based on ultrasound scan
	**Feasibility to identify exposures?**	Linkage to EPD; NIMATS also records medications taken at booking interview (maternal self-report), however this is a free-text variable and prone to variations in spelling. It is also deemed potentially disclosive and therefore not released to the researcher. Upon request, HBS may be able to provide a "dummy" variable for medications of particular interest.<br><br>Limitation: The data relates to primary care prescribing only. Secondary care prescribing data, over the counter medications, and data on adherence to treatments are unavailable using this data source.
	**Feasibility to identify pregnancy outcomes?**	Recorded in NIMATS

For peer review only

Table 5: List of pregnancy outcomes included in this signal detection study from the core outcome set published by the ConcePTION and MuM-PreDiCT consortium

List of core outcomes from ConcePTION consortium	Source of outcome documentation			
	CPRD	SAIL	NIMATS	SMR
Miscarriage	Data available as variable in CPRD Pregnancy Register	Data unavailable<br>Currently, within SAIL researchers are unable to account for terminations as these are classed as sensitive data and not currently accessible for research purposes	Variable recorded in NIMATS, but only if it has occurred after booking appointment (early miscarriages may be under-represented)	Data available in SMR02 when women are admitted to secondary care
Intrauterine death / Stillbirth / perinatal death	Data available as variable in CPRD Pregnancy Register and linked HES data	Data available as stillbirths flagged in NCCH and Annual District Birth Extract (ABDE) datasets	IUD = Yes<br>Stillbirth = Yes<br>Perinatal death = partial (some infant deaths will not be captured in NIMATS but may be picked up in General Register Office (GRO) or (Patient Administration System) PAS data	Data available in SMR02
Small for gestational age (SGA)	Birthweight, baby's sex and gestational age data available within CPRD Pregnancy Register and linked HES data, from which SGA can be derived	Can be derived from birthweight data available in ADBE and NCCH, gestational age available in the latter	Yes – Variable "Birth Centile" available 2016 onwards<br>If missing, can be derived from NIMATS variables (birthweight, sex and gestational age at delivery)	Can be derived from variables recorded in SMR02 (birthweight, sex and gestational age at delivery)
Preterm Birth	Gestational age available within CPRD Pregnancy Register from which preterm birth can be derived	Data available in NCCH	Can be derived from variables recorded in NIMATS (gestational age at delivery, EDC)	Can be derived from variables recorded in SMR02 (gestational age at delivery, EDC)
Overall Congenital anomalies (CA) including termination of pregnancy due to fetal anomaly	Data available in primary care data, and linkage to baby records identified from mother-baby linked (MBL) data	Data available from the Congenital Anomaly Register and Information Service (CARIS) database and the NCCH_SIG_COND dataset which is used to inform it.	Yes – NIMATS (variable =infant complications; value = "congenital abnormality")	Data unavailable
Specific major congenital anomalies (not including termination of pregnancy due to fetal anomaly)	Congenital anomalies recorded with sufficient quality will be included. Data available in primary care data, and linkage to baby records identified from mother-baby linked (MBL) data	Data available in CARIS database	Data unavailable.<br>Data in hospital discharge records are not suitable for identifying specific congenital anomalies.	Data unavailable
Maternal death	Data available in primary care data	Data available from GP/Patient Episode Data for Wales (PEDW)/ADDE for death during childbirth	Data available in GRO	Data available in National Records of Scotland

For peer review only

**Additional list of core outcomes from MuM-PreDiCT consortium**				
**Maternal outcomes**				
**Clinical: antenatal**				
Pre-eclampsia, eclampsia, HELLP syndrome, gestational hypertension	Data available in primary care and linked HES data	Data available in primary care and linked PEDW data (if diagnosed)	Data available in the Delivery details description, Present Pregnancy Problems, and Induction reason variables in NIMATS, as well as diagnoses variable in Patient Administration system (PAS)	Recorded from chapter XV of ICD10 Pregnancy, Childbirth and the Puerperium, (O00-O99).
Placenta abruption	Data available in primary care and linked HES data	Data available in primary care and linked PEDW data (if diagnosed)	Data available in the Delivery details description, Indication for Caesarean section variables in NIMATS, as well as diagnoses variable in Patient Administration system (PAS)	Recorded from chapter XV of ICD10 Pregnancy, Childbirth and the Puerperium, (O00-O99).
Venous thromboembolism	Data available in primary care and linked HES data	Data available in primary care and linked PEDW data (if diagnosed)	Data available as postnatal complications variable in NIMATS and as a diagnoses variable in Patient Administration system (PAS)	Recorded from chapter XV of ICD10 Pregnancy, Childbirth and the Puerperium, (O00-O99).
**Clinical: peripartum**				
Preterm premature rupture of membrane (PPROM)	Data available in linked HES data	Data available in primary care and linked PEDW data (if recorded)	Data available as postnatal complications variable in NIMATS and as a diagnoses variable in Patient Administration system (PAS)	Recorded from chapter XV of ICD10 Pregnancy, Childbirth and the Puerperium, (O00-O99).
Severe maternal morbidity	Specific maternal morbidities recorded with sufficient quality will be included. Data available from primary care data	Data available in primary care and linked PEDW data (if recorded)	Data available as specific maternal morbidities in NIMATS	Recorded from chapter XV of ICD10 Pregnancy, Childbirth and the Puerperium, (O00-O99).
Postpartum haemorrhage (PPH)	Data available in linked HES data	Data available in primary care and linked PEDW data (if recorded)	Data available as postnatal complications variable in NIMATS and as a diagnoses variable in Patient Administration system (PAS)	Recorded from chapter XV of ICD10 Pregnancy, Childbirth and the Puerperium, (O00-O99).
**Mental health**				
Self-harm/suicide	Data available in primary care and linked HES data	Data available in primary care, linked PEDW, and emergency admissions data	Data available in Self Harm and Suicide Ideation Register	Data available in National Records of Scotland
Postpartum mental illness	Data available in primary care and linked HES data	Data available in primary care and linked PEDW data (if diagnosed)	Data available as postnatal complications variable in NIMATS and as a diagnoses variable in Patient Administration system (PAS)	Recorded from chapter XV of ICD10 Pregnancy, Childbirth and the Puerperium, (O00-O99).
**Children's outcomes**				

For peer review only

Cerebral palsy/Autism/ADHD/Neurodevelopmental outcomes	Data available in primary care data, and linkage to baby records identified from mother-baby linked (MBL) data	Data available in primary and linked PEDW data	Partial data available in NI Cerebral Palsy Register (NICPR)	Data unavailable

For peer review only

References

1. Sundari Ravindran TK, Teerawattananon Y, Tannenbaum C, Vijayasingham L. Making pharmaceutical research and regulation work for women. BMJ. 2020 Oct 27;371.
2. Watson S, Caster O, Rochon PA, den Ruijter H. Reported adverse drug reactions in women and men: Aggregated evidence from globally collected individual case reports during half a century. EClinicalMedicine. 2019 Dec 1;17:100188.
3. Figueroa Gray M, Hsu C, Kiel L, Dublin S. Getting through the day: A pilot qualitative study of U.S. women's experiences making decisions about anti-nausea medication during pregnancy. BMC Pregnancy Childbirth. 2018 Dec 4;18(1):1–8.
4. Ngure K, Trinidad SB, Beima-Sofie K, Kinuthia J, Matemo D, Kimemia G, et al. Perceived Social Influences on Women's Decisions to use Medications not Studied in Pregnancy. A Qualitative Ethical Analysis of Preexposure Prophylaxis Implementation Research in Kenya. J Empir Res Hum Res Ethics. 2021 Jun 16;
5. UNIVERSITYoF BIRMINGHAM The Case for UK Leadership in the Development of Safe, Effective and Accessible Medicines for Use in Pregnancy. 2022;
6. UKTIS [Internet]. [cited 2022 Nov 23]. Available from: http://www.uktis.org/
7. Ren Z, Bremer AA, Pawlyk AC. Drug development research in pregnant and lactating women. Am J Obstet Gynecol. 2021 Jul 1;225(1):33–42.
8. Lynch MM, Squiers LB, Kosa KM, Dolina S, Read JG, Broussard CS, et al. Making Decisions About Medication Use During Pregnancy: Implications for Communication Strategies. Matern Child Health J. 2018 Jan 1;22(1):92.
9. Subramanian A, Azcoaga-Lorenzo A, Anand A, Phillips K, Lee SI, Cockburn N, et al. Polypharmacy during pregnancy and associated risk factors: a retrospective analysis of 577 medication exposures among 1.5 million pregnancies in the UK, 2000-2019. BMC Med. 2023;21(1):21.
10. Rochon PA, Petrovic M, Cherubini A, Onder G, O'Mahony D, Sternberg SA, et al. Polypharmacy, inappropriate prescribing, and deprescribing in older people: through a sex and gender lens. Lancet Heal Longev. 2021 May 1;2(5):e290–300.
11. Prevalence of polypharmacy in pregnancy | SAPC [Internet]. [cited 2022 Aug 23]. Available from: https://sapc.ac.uk/conference/2021/abstract/prevalence-of-polypharmacy-pregnancy_p1-29-2
12. Steinberg JR, Turner BE, Weeks BT, Magnani CJ, Wong BO, Rodriguez F, et al. Analysis of Female Enrollment and Participant Sex by Burden of Disease in US Clinical Trials Between 2000 and 2020. JAMA Netw Open. 2021 Jun 1;4(6):e2113749–e2113749.
13. Kaufman DW, Kelly JP, Rosenberg L, Anderson TE, Mitchell AA. Recent Patterns of Medication Use in the Ambulatory Adult Population of the United States: The Slone Survey. JAMA. 2002 Jan 16;287(3):337–44.
14. Huybrechts KF, Bateman BT, Hernández-Díaz S. Use of real-world evidence from healthcare utilization data to evaluate drug safety during pregnancy. Pharmacoepidemiol Drug Saf. 2019 Jul 1;28(7):906–22.
15. Cavadino A, Sandberg L, Öhman I, Bergvall T, Star K, Dolk H, et al. Signal Detection in EUROmediCAT: Identification and Evaluation of Medication-Congenital Anomaly Associations and Use of VigiBase as a Complementary Source of Reference. Drug Saf. 2021 Jul 1;44(7):765–85.
16. Demailly R, Escolano S, Haramburu F, Tubert-Bitter P, Ahmed I. Identifying Drugs Inducing Prematurity by Mining Claims Data with High-Dimensional Confounder

For peer review only

Score Strategies. Drug Saf. 2020 Jun 1;43(6):549–59.
17. Cederholm S, Hill G, Asiimwe A, Bate A, Bhayat F, Persson Brobert G, et al. Structured Assessment for Prospective Identification of Safety Signals in Electronic Medical Records: Evaluation in the Health Improvement Network. Drug Saf. 2015 Jan 22;38(1):87.
18. Final Report Summary - EUROMEDICAT (EUROmediCAT: Safety of Medication use in Pregnancy in Relation to Risk of Congenital Malformations) | FP7 | CORDIS | European Commission [Internet]. [cited 2022 Nov 24]. Available from: https://cordis.europa.eu/project/id/260598/reporting
19. Herrett E, Gallagher AM, Bhaskaran K, Forbes H, Mathur R, Staa T van, et al. Data Resource Profile: Clinical Practice Research Datalink (CPRD). Int J Epidemiol. 2015 Jun 1;44(3):827–36.
20. CPRD GOLD July 2022 dataset | CPRD [Internet]. [cited 2022 Nov 26]. Available from: https://cprd.com/cprd-gold-july-2022-dataset
21. Minassian C, Williams R, Meeraus WH, Smeeth L, Campbell OMR, Thomas SL. Methods to generate and validate a Pregnancy Register in the UK Clinical Practice Research Datalink primary care database. Pharmacoepidemiol Drug Saf. 2019 Jul 1;28(7):923–33.
22. Jones KH, Ford D V., Thompson S, Lyons RA. A Profile of the SAIL Databank on the UK Secure Research Platform. Int J Popul Data Sci. 2019 Nov 20;4(2).
23. Lee SI, Azcoaga-Lorenzo A, Agrawal U, Kennedy JI, Fagbamigbe AF, Hope H, et al. Epidemiology of pre-existing multimorbidity in pregnant women in the UK in 2018: a population-based cross-sectional study. BMC Pregnancy Childbirth. 2022 Dec 1;22(1):1–15.
24. Given J, Casson K, Dolk H, Loane M. Evaluation of maternal medications and diseases recorded in Northern Ireland Maternity System (NIMATS) database compared to NI Enhanced Prescribing Database (EPD): a data linkage validation study. Int J Popul Data Sci. 2018 Jun 11;3(2).
25. Lee SI, Eastwood KA, Moss N, Azcoaga-Lorenzo A, Subramanian A, Anand A, et al. Protocol for the development of a core outcome set for studies of pregnant women with pre-existing multimorbidity. BMJ Open. 2021 Oct 1;11(10):e044919.
26. Damase-Michel C, Wurst K. Core evidence elements for generating medication safety evidence for pregnancy using population-based data (D1.2). 2021 Jul 5;
27. Welsh Dispensing Dataset (WDDS) [Internet]. [cited 2022 Aug 23]. Available from: https://web.www.healthdatagateway.org/dataset/50ef6443-ed4b-40f9-97fb-1cfd53be6579
28. 12 Weeks Pregnant | Pregnancy | Start for Life [Internet]. [cited 2023 Feb 14]. Available from: https://www.nhs.uk/start4life/pregnancy/week-by-week/1st-trimester/week-12/#anchor-tabs
29. A healthy lifestyle - WHO recommendations [Internet]. [cited 2023 Jul 11]. Available from: https://www.who.int/europe/news-room/fact-sheets/item/a-healthy-lifestyle---who-recommendations
30. Gail MH, Altman DG, Cadarette SM, Collins G, Evans SJW, Sekula P, et al. Design choices for observational studies of the effect of exposure on disease incidence. BMJ Open. 2019 Dec 9;9(12).
31. Avillach P, Salvo F, Thiessard F, Miremont-Salamé G, Fourrier-Reglat A, Haramburu F, et al. Pilot evaluation of an automated method to decrease false-positive signals induced by co-prescriptions in spontaneous reporting databases. Pharmacoepidemiol Drug Saf. 2014 Feb 1;23(2):186–94.

For peer review only

Supplementary Table 1: Operational definition of pregnancy outcomes included in this signal detection study

Outcomes	Operational definition	Observation time window
**Miscarriage**	(1) Diagnostic code in primary care OR<br>(2) Diagnostic code in secondary care OR<br>(3) Database specific flag for miscarriage	Diagnostic code or flag recorded until 30 weeks of pregnancy (includes 6-week lag period for recording)
**Intrauterine death / Stillbirth / perinatal death**	(1) Diagnostic code in primary care OR<br>(2) Diagnostic code in secondary care OR<br>(3) Database specific flag for IUD/Stillbirth/perinatal death	Diagnostic code or flag recorded from 24 weeks of pregnancy until six weeks after delivery date (includes 6-week lag period for recording)
**Small for gestational age (SGA)**	(1) Diagnostic code in primary care OR<br>(2) Diagnostic code in secondary care OR<br>(3) Database specific flag for SGA OR<br>(4) Database specific continuous variable for gestational age, sex and birthweight (indicating birthweight below the 10th percentile for babies of the same gestational age according to the INTERGROWTH-21st birth weight standard)	Diagnostic code or flag recorded +/- 6 weeks from delivery date
**Preterm birth**	(1) Diagnostic code in primary care OR<br>(2) Diagnostic code in secondary care OR<br>(3) Database specific flag for preterm birth OR<br>(4) Database specific continuous variable for gestational age (indicating gestational age between 22 to 37 weeks)<br>(5)	Diagnostic code or flag recorded +/- 6 weeks from delivery date
**Overall Congenital anomalies (CA)**	(1) Diagnostic code in primary care OR<br>(2) Diagnostic /operational code in secondary care OR<br>(3) Database specific flag for congenital anomalies	Diagnostic code or flag recorded in the baby's medical records until 1 year after birth (delivery date)
**Specific major congenital anomalies**	(1) Diagnostic code in primary care OR<br>(2) Diagnostic code in secondary care OR<br>(3) Database specific flag for congenital anomalies	Diagnostic code or flag recorded in the baby's medical records until 1 year after birth (delivery date)
**Maternal death**	(1) Death flag in primary care OR<br>(2) Death flag in the linked death registry	Death flag recorded until 1 year after pregnancy end date

For peer review only

**Pre-eclampsia, eclampsia, HELLP syndrome, gestational hypertension**	(1) Diagnostic code in primary care OR<br>(2) New primary care prescription of antihypertensives during pregnancy OR<br>(3) Diagnostic code in secondary care OR<br>(4) Database specific flag for pre-eclampsia/HELLP syndrome/gestational hypertension<br><br>Exclusion: Patients with a pre-existing record of chronic hypertension or antihypertensives prior to pregnancy or within 20 weeks of pregnancy	Diagnostic code recorded from 24 weeks of pregnancy until six weeks after pregnancy end date (includes 6-week lag period for recording)
**Placenta abruption**	(1) Diagnostic code in primary care OR<br>(2) Diagnostic code in secondary care OR<br>(3) Database specific flag for placenta abruption	Diagnostic code or flag recorded until 6 weeks after pregnancy end date (includes 6-week lag period for recording)
**Venous thromboembolism**	(1) Diagnostic code in primary care OR<br>(2) Diagnostic code in secondary care OR<br>(3) Database specific flag for venous thromboembolism	Diagnostic code or flag recorded until 12 weeks after pregnancy end date (includes 6-week lag period for recording)
**Preterm premature rupture of membrane (PPROM)**	(1) (Diagnostic code for premature rupture of membrane in primary care OR<br>(2) Diagnostic code for premature rupture of membrane in secondary care OR<br>(3) Database specific flag for premature rupture of membrane)<br><br>AND<br><br>Operational definition met for premature birth	Diagnostic code or flag recorded +/- 6 weeks from delivery date
**Severe maternal morbidity**	(1) Diagnostic code in primary care OR<br>(2) Diagnostic /operational code in secondary care for one of the following morbidities<br><br>1. Acute myocardial infarction<br>2. Aneurysm<br>3. Acute Renal Failure<br>4. Adult respiratory distress syndrome<br>5. Amniotic fluid embolism<br>6. Cardiac arrest or ventricular fibrillation<br>7. Disseminated intravascular coagulation<br>8. Eclampsia	Diagnostic code recorded until 3 months after pregnancy end date

For peer review only

	9. Heart failure or arrest during surgery or procedure<br>10. Puerperal cerebrovascular disorders<br>11. Pulmonary oedema or Acute heart failure<br>12. Severe anaesthesia complications<br>13. Sepsis<br>14. Shock<br>15. Sickle cell disease with crisis<br>16. Air and thrombotic embolism<br>17. Conversion of cardiac rhythm<br>18. Blood products transfusion<br>19. Hysterectomy<br>20. Temporary tracheostomy<br>21. Ventilation	
**Postpartum haemorrhage (PPH)**	(1) Diagnostic code in primary care OR<br>(2) Diagnostic /operational code in secondary care	Diagnostic code recorded until 3 months after pregnancy end date
**Self-harm/suicide**	(1) Diagnostic code in primary care OR<br>(2) Diagnostic /operational code in secondary care OR<br>(3) Suicide as cause of death in the linked death registry	Diagnostic code or death flag recorded until 1 year after pregnancy end date
**Postpartum mental illness**	(1) Diagnostic code in primary care OR<br>(2) Diagnostic code in secondary care	Diagnostic code recorded until 1 year after pregnancy end date
**Cerebral palsy/Autism/ADHD/Neurodevelopmental outcomes**	(1) Diagnostic code in primary care OR<br>(2) Diagnostic code in secondary care	Diagnostic code recorded anytime in the baby's medical records

For peer review only

Supplementary Table 2: The RECORD statement – checklist of items, extended from the STROBE statement, that should be reported in observational studies using routinely collected health data.

	Item No.	STROBE items	Location in manuscript where items are reported	RECORD items	Location in manuscript where items are reported
**Title and abstract**					
	1	(a) Indicate the study's design with a commonly used term in the title or the abstract (b) Provide in the abstract an informative and balanced summary of what was done and what was found		RECORD 1.1: The type of data used should be specified in the title or abstract. When possible, the name of the databases used should be included.	Multiple databases are planned to be used. This is mentioned in methods and analysis section of the abstract.
				RECORD 1.2: If applicable, the geographic region and timeframe within which the study took place should be reported in the title or abstract.	Mentioned in the methods and analysis section of the abstract.
				RECORD 1.3: If linkage between databases was conducted for the study, this should be clearly stated in the title or abstract.	No linkage involved in the study
**Introduction**					
Background rationale	2	Explain the scientific background and rationale for the investigation being reported			Introduction section, para 4
Objectives	3	State specific objectives, including any prespecified hypotheses			Aims section
**Methods**					
Study Design	4	Present key elements of study design early in the paper			Study design section within

For peer review only

					methods and analysis
Setting	5	Describe the setting, locations, and relevant dates, including periods of recruitment, exposure, follow-up, and data collection			Table 1
Participants	6	*(a) Cohort study* - Give the eligibility criteria, and the sources and methods of selection of participants. Describe methods of follow-up<br>*Case-control study* - Give the eligibility criteria, and the sources and methods of case ascertainment and control selection. Give the rationale for the choice of cases and controls<br>*Cross-sectional study* - Give the eligibility criteria, and the sources and methods of selection of participants<br><br>*(b) Cohort study* - For matched studies, give matching criteria and number of exposed and unexposed<br>*Case-control study* - For matched studies, give matching criteria and the number of controls per case		RECORD 6.1: The methods of study population selection (such as codes or algorithms used to identify subjects) should be listed in detail. If this is not possible, an explanation should be provided.<br><br>RECORD 6.2: Any validation studies of the codes or algorithms used to select the population should be referenced. If validation was conducted for this study and not published elsewhere, detailed methods and results should be provided.<br><br>RECORD 6.3: If the study involved linkage of databases, consider use of a flow diagram or other graphical display to demonstrate the data linkage process, including the number of individuals with linked data at each stage.	Table 1<br><br>Reference number 23<br><br>No linkage
Variables	7	Clearly define all outcomes, exposures, predictors, potential confounders, and effect modifiers. Give diagnostic criteria, if applicable.		RECORD 7.1: A complete list of codes and algorithms used to classify exposures, outcomes, confounders, and effect modifiers should be provided. If these cannot be reported, an explanation should be provided.	This is a signal detection study with a wide range of exposures and outcomes.
Data sources/ measurement	8	For each variable of interest, give sources of data and details			Table 2

For peer review only

		of methods of assessment (measurement). Describe comparability of assessment methods if there is more than one group			
Bias	9	Describe any efforts to address potential sources of bias			Systematic signal review section under methods and analysis
Study size	10	Explain how the study size was arrived at			Table 1
Quantitative variables	11	Explain how quantitative variables were handled in the analyses. If applicable, describe which groupings were chosen, and why			Exposure section, paragraph 2 and covariates section under methods and analysis
Statistical methods	12	(a) Describe all statistical methods, including those used to control for confounding<br><br>(b) Describe any methods used to examine subgroups and interactions<br>(c) Explain how missing data were addressed<br><br>(d) *Cohort study* - If applicable, explain how loss to follow-up was addressed<br>*Case-control study* - If applicable, explain how matching of cases and controls was addressed<br>*Cross-sectional study* - If applicable, describe analytical methods taking account of sampling strategy			(a) Statistical Analysis section, para 2 under methods and analysis<br>(b) No subgroups<br><br>(c) Covariates section under methods and analysis<br>(d) No matching

For peer review only

		(e) Describe any sensitivity analyses			(e) No sensitivity analyses
Data access and cleaning methods		..		RECORD 12.1: Authors should describe the extent to which the investigators had access to the database population used to create the study population.<br><br>RECORD 12.2: Authors should provide information on the data cleaning methods used in the study.	Table 1<br><br>Described elsewhere (Reference 23)
Linkage		..		RECORD 12.3: State whether the study included person-level, institutional-level, or other data linkage across two or more databases. The methods of linkage and methods of linkage quality evaluation should be provided.	No linkage
**Results**					
Participants	13	(a) Report the numbers of individuals at each stage of the study (*e.g.*, numbers potentially eligible, examined for eligibility, confirmed eligible, included in the study, completing follow-up, and analysed)<br>(b) Give reasons for non-participation at each stage.<br>(c) Consider use of a flow diagram		RECORD 13.1: Describe in detail the selection of the persons included in the study (*i.e.*, study population selection) including filtering based on data quality, data availability and linkage. The selection of included persons can be described in the text and/or by means of the study flow diagram.	Study population section under methods and analysis
Descriptive data	14	(a) Give characteristics of study participants (*e.g.*, demographic, clinical, social) and information on exposures and potential confounders<br>(b) Indicate the number of participants with missing data for each variable of interest			This is a protocol paper

For peer review only - http://bmjopen.bmj.com/site/about/guidelines.xhtml

		(c) *Cohort study* - summarise follow-up time (*e.g.*, average and total amount)			
Outcome data	15	*Cohort study* - Report numbers of outcome events or summary measures over time<br>*Case-control study* - Report numbers in each exposure category, or summary measures of exposure<br>*Cross-sectional study* - Report numbers of outcome events or summary measures			This is a protocol paper
Main results	16	(a) Give unadjusted estimates and, if applicable, confounder-adjusted estimates and their precision (e.g., 95% confidence interval). Make clear which confounders were adjusted for and why they were included<br>(b) Report category boundaries when continuous variables were categorized<br>(c) If relevant, consider translating estimates of relative risk into absolute risk for a meaningful time period			This is a protocol paper
Other analyses	17	Report other analyses done—e.g., analyses of subgroups and interactions, and sensitivity analyses			This is a protocol paper
**Discussion**					
Key results	18	Summarise key results with reference to study objectives			This is a protocol paper
Limitations	19	Discuss limitations of the study, taking into account sources of potential bias or imprecision.		RECORD 19.1: Discuss the implications of using data that were not created or collected to answer the specific research question(s). Include	Systematic Signal review section under methods and analysis

For peer review only

		Discuss both direction and magnitude of any potential bias		discussion of misclassification bias, unmeasured confounding, missing data, and changing eligibility over time, as they pertain to the study being reported.	
Interpretation	20	Give a cautious overall interpretation of results considering objectives, limitations, multiplicity of analyses, results from similar studies, and other relevant evidence			This is a protocol paper
Generalisability	21	Discuss the generalisability (external validity) of the study results			This is a protocol paper
**Other Information**					
Funding	22	Give the source of funding and the role of the funders for the present study and, if applicable, for the original study on which the present article is based			Funding section
Accessibility of protocol, raw data, and programming code		..		RECORD 22.1: Authors should provide information on how to access any supplemental information such as the study protocol, raw data, or programming code.	This is a protocol paper

*Reference: Benchimol EI, Smeeth L, Guttmann A, Harron K, Moher D, Petersen I, Sørensen HT, von Elm E, Langan SM, the RECORD Working Committee. The REporting of studies Conducted using Observational Routinely-collected health Data (RECORD) Statement. *PLoS Medicine* 2015; in press.

*Checklist is protected under Creative Commons Attribution (CC BY) license.

For peer review only - http://bmjopen.bmj.com/site/about/guidelines.xhtml

BMJ Open

Detection and evaluation of signals associated with exposure to individual and combination of medications in pregnancy: A signal detection study protocol

Journal:	*BMJ Open*
Manuscript ID	bmjopen-2023-073162.R2
Article Type:	Protocol
Date Submitted by the Author:	14-Sep-2023

Complete List of Authors:	Subramanian, Anuradhaa; University of Birmingham, Institute of Applied Health Research; AstraZeneca UK Limited<br>Lee, Siang Ing; University of Birmingham Institute of Applied Health Research<br>Hemali Sudasinghe, Sudasing Pathirannehelage Buddhika; University of Birmingham, Institute of Applied Health Research<br>Wambua, Steven; University of Birmingham, Applied health research<br>Phillips, Katherine; University of Birmingham, Applied health research<br>Singh, Megha; University of Birmingham, Applied health research<br>Azcoaga-Lorenzo, Amaya; University of Saint Andrews School of Medicine, Division of Population and Behavioural Sciences; Instituto de Investigación Sanitaria Fundación Jimenez Diaz, Hospital Rey Juan Carlos. Research Network on Chronicity, Primary Care and Health Promotion-RICAPPS (RICORS)<br>Cockburn, Neil; University of Birmingham, Applied health research<br>Wang, Jingya; Institute of Applied Health Research University of Birmingham<br>Fagbamigbe, Adeniyi; University of St Andrews, PBS; University of Ibadan, EMS<br>Usman, Muhammad; University of Ibadan, University of St Andrews<br>Damase-Michel, Christine; Service de Pharmacologie Médicale, CHU Toulouse, Université Toulouse III<br>Yau, Christopher; The University of Manchester, Division of Informatics, Imaging and Data Sciences, Faculty of Biology Medicine and Health<br>Kent, Lisa; Queen's University Belfast, Centre for Public Health<br>McCowan, Colin ; University of St. Andrews<br>OReilly, Dermot; Queens University Belfast, Epidemiology and Public Health<br>Santorelli, Gillian; Bradford Teaching Hospitals NHS Foundation Trust<br>Hope, Holly; Manchester University<br>Kennedy, Jonathan; Swansea University, College of Medicine<br>Mhereeg, Mohamed; Swansea University Medical School<br>Abel, Kathryn ; Institute of Brain Behaviour and Mental Health, Medical and Human Sciences; Manchester Mental Health & Social Care Trust<br>Eastwood, Kelly-Ann; University Hospitals Bristol and Weston NHS Foundation Trust<br>Black, Mairead; University of Aberdeen<br>Loane , Maria ; University of Ulster<br>Moss, Ngawai; Queen Mary University of London

	Brophy, Sinead; Swansea University, College of Medicine<br>Brocklehurst, Peter; University of Birmingham<br>Dolk, Helen; Ulster University - Jordanstown Campus, Institute for Nursing and Health Research<br>Nelson-Piercy, Catherine; Guy’s & St Thomas’ Foundation Trust<br>Nirantharakumar, Krishnarajah; University of Birmingham
**Primary Subject Heading**:	Reproductive medicine
Secondary Subject Heading:	Epidemiology, Obstetrics and gynaecology, Public health
Keywords:	OBSTETRICS, Maternal medicine < OBSTETRICS, EPIDEMIOLOGY

SCHOLARONE™
Manuscripts

Detection and evaluation of signals associated with exposure to individual and combination of medications in pregnancy: A signal detection study protocol

Authors: Anuradhaa Subramanian[1], Siang Ing Lee[1], Sudasing Pathirannehelage Buddhika Hemali Sudasinghe[1], Steven Wambua[1], Katherine Phillips[1], Megha Singh[1], Amaya Azcoaga-Lorenzo[2,3], Neil Cockburn[1], Jingya Wang[1], Adeniyi Francis Fagbamigbe[2,4], Muhammad Usman[3], Christine Damase-Michel[5,6], Christopher Yau[7,8], Lisa Kent[9], Colin McCowan[2], Dermot O'Reilly[9], Gillian Santorelli[10], Holly Hope[11], Jonathan I Kennedy[12], Mohamed Mhereeg[12], Kathryn M Abel[11,13], Kelly-Ann Eastwood[9, 14], Mairead Black[15], Maria Loane[16], Ngawai Moss[17], Sinead Brophy[12], Peter Brocklehurst[1#], Helen Dolk[18#], Catherine Nelson-Piercy[19#], Krishnarajah Nirantharakumar[1#], on behalf of the MuM-PreDiCT Group.

[#] joint senior authors

Corresponding author: Prof Krishnarajah Nirantharakumar
k.nirantharan@bham.ac.uk

Affiliation:

1. Institute of Applied Health Research, University of Birmingham, Birmingham, UK
2. Division of Population and Behavioural Sciences, School of Medicine, University of St Andrews, UK
3. Hospital Rey Juan Carlos, Instituto Investigacion Sanitaria Fundacion Jimenez Diaz
4. Department of Epidemiology and Medical Statistics, College of Medicine, University of Ibadan, Nigeria
5. Medical and Clinical Pharmacology, School of Medicine, Université Toulouse III, France
6. INSERM, Center for Epidemiology and Research in Population Health (CERPOP), France
7. Division of Informatics, Imaging and Data Sciences, Faculty of Biology Medicine and Health, The University of Manchester, UK
8. Health Data Research UK
9. Centre for Public Health, Queen's University of Belfast, UK
10. Bradford Institute for Health Research, UK
11. Centre for Women's Mental Health, Division of Psychology and Mental Health, School of Health Sciences, Faculty of Biology Medicine & Health, The University of Manchester, UK
12. Data Science, Medical School, Swansea University, UK
13. Greater Manchester Mental Health NHS Foundation Trust, Manchester, UK
14. St Michael's Hospital, University Hospitals Bristol NHS Foundation Trust, UK
15. Aberdeen Centre for Women's Health Research, School of Medicine, Medical Science and Nutrition, University of Aberdeen, UK
16. The Institute of Nursing and Health Research, Ulster University, UK
17. Patient and public representative, London, UK
18. School of Medicine, Ulster University, UK.
19. Guy's and St. Thomas' NHS Foundation Trust, UK

For peer review only

Abstract

Introduction

Considering the high prevalence of polypharmacy in pregnant women and the knowledge gap in the risk-benefit safety profile of their often-complex treatment plan, more research is needed to optimise prescribing. In this study, we aim to detect adverse and protective effect signals of exposure to individual and pairwise combinations of medications during pregnancy.

Methods and analysis

Using a range of real-world data sources from the United Kingdom (UK), we aim to conduct a pharmacovigilance study to assess the safety of medications prescribed during the preconception period (three months prior to conception) and first trimester of pregnancy. Women aged between 15-49 years with a record of pregnancy within the Clinical Practice Research Datalink (CPRD) Pregnancy Register, the Welsh Secure Anonymised Information Linkage (SAIL), the Scottish Morbidity Record datasets (SMR), and the Northern Ireland Maternity System (NIMATS) will be included.

A series of case control studies will be conducted to estimate measures of disproportionality, detecting signals of association between a range of pregnancy outcomes and exposure to individual and combinations of medications.

A multidisciplinary expert team will be invited to a signal detection workshop. By employing a structured framework, signals will be transparently assessed by each member of the team using a questionnaire appraising the signals on aspects of temporality, selection, time, and measurement related biases and confounding by underlying disease or co-medications. Through group discussion, the expert team will reach consensus on each of the medication exposure-outcome signal, thereby excluding spurious signals, leaving signals suggestive of causal associations for further evaluation.

Ethics and dissemination

Ethical approval has been obtained from the Independent Scientific Advisory Committee, SAIL Information Governance Review Panel, University of St. Andrews Teaching and Research Ethics Committee and Office for Research Ethics Committees Northern Ireland (ORECNI) for access and use of CPRD, SAIL, SMR and NIMATS data respectively.

For peer review only

Strengths and limitations of this study

- A comprehensive range of medication exposures prescribed both individually and as a combination with another medication within primary care is included in this study, providing a wide opportunity to detect and evaluate signals of adverse and protective effect during pregnancy
- This study utilises a wide range of data sources obtaining prescription data from primary care (from Clinical Practice Research Datalink and Secure Anonymized Information Linkage), and the community (from Prescribing Information System), but is limited by the unavailability of prescription data from secondary care
- The results of this signal detection study are limited by the quality of routinely collected exposure and outcome data used for this study
- This signal detection study is susceptible to type 1 and type 2 errors owing to limitations in terms of multiple testing and insufficient sample size respectively
- While the results of this exploratory signal detection study may provide useful signals, they may suffer from biases related to confounding, and must be followed up by methodologically rigorous pharmacoepidemiological studies focussed on each signal separately

For peer review only

Introduction

Conventional methods of drug development from discovery to pre-clinical and clinical research and review are expensive, time consuming and disproportionately concentrate on disease areas that predominantly affect men.(1) The effect of this can be seen in the female dominance of adverse drug effect reporting, especially during the period of reproductive age.(2) Pregnant and breast-feeding women are further inclined to become `therapeutic orphans', by their routine exclusion from clinical trials, given the struggle to insure such trials involving pregnant women. Pregnant women are further disadvantaged by having to arduously navigate through multifarious social influences, some losing their autonomy in medical decision making around taking medications or in their choice to participate in a clinical trial during pregnancy.(3,4)

In light of the urgent action required to develop safe medications for use during pregnancy, the 'Healthy Mum, Healthy Baby, Healthy Future' report by the Birmingham Health Partners advocates inclusion of pregnant women in clinical trials unless there are specific safety concerns.(5) The report also makes a case to pilot a 'Maternal Investigation Plan' similar to the 'Paediatric Investigation Plan' implemented by European Union, to make licensing compulsory for use of medications during pregnancy.

In the absence of safety signals for medication exposure during pregnancy from clinical trial data, information on teratogenicity is limited, and is only prospectively collected from national surveillance data, available from resources such as UK Teratology Information Service (UKTIS).(6) This limited availability of robust evidence on medication use during pregnancy has led to women often being prescribed medications 'off-label' in practice.(7) Without appropriate medication safety information, the decision to continue, discontinue or switch their medication falls to the women themselves and their healthcare providers.(8)

We have previously found that the prevalence of polypharmacy in the United Kingdom (UK), during the first trimester of pregnancy alone, has increased from 8.7% to 18.7% over the last two decades.(9) While polypharmacy itself may be essential and beneficial in the management of multiple chronic conditions in combination with pregnancy related complications, inappropriate polypharmacy may cause preventable adverse drug events (ADEs) due to drug-drug interactions.(10) Considering the high prevalence of polypharmacy among women of reproductive age and the knowledge gap in safety profiles of complex treatment plan,(9,11–13) more research is urgently needed to optimise prescribing by detecting signals of adverse outcomes following exposure to combination of medications.

Real world data (RWD) can be used to assess the post-market safety of the use of individual and combinations of medication during pregnancy, generating evidence to support regulatory decision making. In addition, RWD also provides an opportunity for repurposing of approved medications as prophylactic treatments for pregnancy related complications.(14) Real world data-based signal detection involves a systematic step-by-step approach(15–17): (1) identifying real world data source and inspecting its feasibility in accurately ascertaining exposures and outcomes to support identification of safety signals, (2) listing the outcomes (adverse events) and exposures (medications) of interest, (3) generating measures of statistical association for large sets of exposure-outcome pairs with methodological design to limit confounding, (4) reviewing identified signals by a multidisciplinary team and considering sources of bias that lead to false positive signals to ensure contextual interpretation and (5)

For peer review only

strengthening and confirming screened signals using rigorous pharmaco-epidemiological studies or clinical trials.

The feasibility of mining real world data to detect adverse and protective effect signals of individual medications during pregnancy has already been established.(16,18) In this study, as part of the "Multimorbidity and Pregnancy: Determinants, Clusters, Consequences and Trajectories (MuM-PreDiCT)" consortium, we aim to develop and implement a systematic signal detection methodology to determine adverse and protective signals from both individual and combinatorial medication exposure on the incidence of various pregnancy outcomes, using a set of large primary care, secondary care and maternity care databases within the UK.

Aims

The study aims to evaluate the feasibility of conducting a signal detection study estimating and evaluating the adverse and protective signals of medications prescribed during the preconception period and the first trimester of pregnancy on the incidence of various pregnancy outcomes using real world data

The key objectives include:

(1) To scope real world data sources in UK, and to check their feasibility for signal detection by exploring the range of medications prescribed and recorded within said data sources during the preconception period and first trimester of pregnancy
(2) To identify a core list of pregnancy outcomes, and to explore the feasibility of capturing these outcomes within said data sources
(3) To apply suitably developed methods from published literature to estimate measures of disproportionality for each of the exposure-outcome pairs and detect signals
(4) To systematically review the detected signals through a multidisciplinary expert committee workshop, using a pre-specified structured questionnaire exploring sources of false positives.

Methods and analysis

Data source

Four population-based data sources spanning all four nations of the UK will provide data for this signal detection study. These include primary care data sources such as Clinical Practice Research Datalink (CPRD, from all four nations) and The Secure Anonymized Information Linkage (SAIL, Wales) and secondary care data sources such as Hospital Episode Statistics (HES, England), Scottish Morbidity Records (SMR) with linked community prescription data (Scotland), and Northern Ireland Maternity System (NIMATS) with linked Enhanced Prescribing Database (EPD, Northern Ireland). The data sources are scoped, and their feasibility for this signal detection study is assessed and tabulated in Table 1-4.

(1) Clinical Practice Research Datalink and Hospital Episode Statistics

CPRD Gold and Aurum contains anonymized, longitudinal medical records of over 20 and 39 million patients in the UK collected by over 985 and 1,489 participating general practices

For peer review only

respectively as part of their care and support.(19) It currently covers general practices that use the Vision and EMIS software, and collects data from 20% of general practices in the UK.(20) It includes data on demographics, diagnoses and prescriptions. Linkage to area-based deprivation index known as the Index of Multiple Deprivation (IMD) and Hospital Episodes Statistics (HES) is available for 75% of patients in England, whose general practices have consented to the CPRD linkage scheme.

CPRD Gold contains data on medications prescribed within primary care with associated prescription time stamps, encoded using drug codes that are assigned and categorized according to British National Formulary (BNF) item codes. However, CPRD Gold may be limited by their unavailability of secondary care and over the counter medication data, and data on whether these prescriptions were dispensed.

Within CPRD, the CPRD Pregnancy Register is an algorithm that takes information from maternity, antenatal and birth health records from primary care to detect pregnancies and their outcomes.(21) A subset of the Pregnancy Register, Mother Baby Linked (MBL) data further allows for studying outcomes in the children.

(2) The Secure Anonymized Information Linkage

The SAIL databank, a population level database in Wales, is a repository of anonymized health and socio-economic administrative data that provide linkage at an individual level.(22) It holds health data for 4.8 million people and includes data contributed by 80% of Welsh general practices. National Community Child Health Dataset, GP records and hospital records have been used to detect pregnancies in SAIL.(23) In addition, patient level linkage to the Welsh Longitudinal General Practice dataset and the Welsh Demographic Service dataset has been used to obtain data on diagnoses, prescriptions and demographics data respectively. The Welsh Dispensing Data Set (WDDS) containing information on general practitioner (GP) prescribed medications and dispensed medications by community contractors has been linked to the SAIL databank. Similar to CPRD, SAIL may be limited by its unavailability of secondary care and over the counter medication data and data on whether the prescriptions were dispensed.

(3) Scottish Maternity Records and linked data sources

The Scottish Maternity Records (SMR02) will be linked to data from Hospital Admissions (SMR01), Mental Health Inpatients (SMR04), Accident and Emergency and the Demography and Death registries,(23) covering diagnoses and demographic data for all inpatient stays and day cases for residents in Scotland. Maternity records (SMR02) or pregnancy-related hospital admissions (SMR01) allows for identification of pregnancies, and prescription data of medications dispensed in the community can be obtained from linked Prescribing Information System (PIS).

(4) Northern Ireland Maternity System

NIMATs holds demographic and clinical information on mothers and infants. It captures data relating to the current complete maternity process, but also contains details about the mother's past medical and obstetric history. NIMATs contains information on medications that mothers self-reported to have taken during pregnancy. In addition to self-reported data on

For peer review only

medications, linkage of NIMATs to EPD allows for analysis of prescription data issued by GPs.(24)

Study design

A series of case control studies will be conducted to estimate measures of disproportionality, detecting signals of association between each of the pregnancy outcomes of interest and exposure to individual or combination of medications prescribed during the preconception period and first trimester of pregnancy.

Study population

For this feasibility study, we aim to conduct the analyses separately across all four databases described in the section above. Women aged between 15-49 years with a pregnancy recorded within the CPRD Gold Pregnancy Register (all four nations), National Community Child Health data (Wales), SMR02 (Scotland), and NIMATS (Northern Ireland), within the study period customized to the period of data availability within each data source will be eligible for inclusion in this study (Table 1-4). Data standard quality checks for each of the databases, and eligibility criteria for inclusion is presented in a previous publication.(23) Pregnancy start dates will be either derived using a pre-defined algorithm or used as reported within the said data source (Table 1-4), and will be used to define exposure and outcome time windows.

Outcomes

The MuM-PreDiCT and ConcePTION consortium have developed core outcome sets (a minimum set of recommended maternal and offspring outcomes) for studies of pregnant women with multiple long-term conditions and for studies generating medication safety evidence respectively.(25,26) The outcome set was reviewed by a study advisory group panel comprising of general practitioners, obstetricians, obstetric physicians and experienced users of the available data sources, to identify the pregnancy outcomes' suitability and feasibility for inclusion in this signal detection study. The availability, prevalence, quality, and completeness of data recording of these outcomes within the said data sources were used as criteria to determine the feasibility of the outcome of interest. Outcomes with poor recording in a specific database may not be included in the analysis using that database to avoid noisy signals due to insufficient power or case misclassification. Furthermore, outcomes that cannot be considered as a medication adverse event such as termination of pregnancy, outcomes that were too broad or non-specific such as involvement of patients in care decisions, and neonatal outcomes that were too narrow and reflective of prematurity such as intubation/ventilation requirement were excluded. The final list of outcomes to be considered for inclusion in this study is available in Table 5, and the operational definitions of these outcomes is available in Supplementary Table 1.

Exposure

All medications prescribed within primary care [as in the CPRD Gold database (all four nations) and EPD (Northern Ireland)] and all medications dispensed in the community [as in the PIS database (Scotland)] have a BNF code associated with them in the UK. Dispensed medications in WDDS (Wales) are coded based on the Dictionary of Medicines and Devices (DM+D). Using a complete extract of the dictionary,(27) each dispensed item within the dictionary can be mapped to a corresponding BNF code.

For peer review only - http://bmjopen.bmj.com/site/about/guidelines.xhtml

Analysis stratified by BNF codes has been established in a previous analysis, wherein using a dictionary of medications prescribed within primary care , we stratified the medications prescribed according to their 4-digit BNF code (BNF chapter, section, paragraph, and sub-paragraph), screened and selected 577 BNF items that were pharmacological agents with therapeutic action.(9)

Using a similar strategy, we will ascertain the exposure information for a range of medications stratified by their BNF code specifically within four crucial time windows: (1) preconception period (up to 90 days prior to the start of pregnancy) (2) first trimester of pregnancy (first 12 weeks of pregnancy), (3) second trimester of pregnancy (between 13 and 26 weeks of pregnancy), and (4) third trimester of pregnancy (between 27 weeks and end of pregnancy) . (28) However, the exposure ascertainment within these windows will be restricted further to the time prior to outcome diagnosis to preserve exposure-outcome temporality. For outcomes that occur during the first trimester of pregnancy such as miscarriage, the exposure time window will be restricted to the preconception period and first trimester only. Furthermore, we will ascertain the exposure information for a range of medication pairs that are prescribed concurrently within the same exposure window to assess adverse and protective effect signals associated with medications prescribed in pairs.

Covariates

The following demographic and health characteristics will be obtained from the four datasets; maternal age at the start of pregnancy, ethnicity, smoking status as recorded prior to the start of pregnancy, pre-gravid body mass index (BMI) and a wide range of comorbidities. Patients with missing data on smoking status, pre-gravid BMI, and ethnicity will be categorized into a separate missing category within the corresponding variable. The list of 79 pre-existing long-term comorbidities for which baseline data were extracted, and their definitions is presented in a previous publication. (23) Ethnicity will be categorized as White, South Asian, Black Afro-Caribbean, mixed ethnic background, and other ethnic minority groups including Chinese. Latest BMI recorded prior to the start of pregnancy will be considered as pre-gravid BMI, and will be categorized according to the World Health Organization definition as underweight (<18.5 kg/m^2), healthy weight (18.5-24.9 kg/m^2), overweight (25-29.9 kg/m^2), and obese (≥30 kg/m^2).(29) Smoking status of patients prior to the start of pregnancy will categorise patients as current smokers, ex-smokers, and never-smokers.

For peer review only

Statistical analysis

Description of baseline characteristics

Patient covariates will be summarized and stratified by their outcome status using numbers and percentages for categorical variables and mean (standard deviation) or median (interquartile range) for continuous variables.

Statistical analysis

Odds ratio (OR) will be considered as the primary measure of disproportionality, and will be estimated for each of the exposure-outcome pairs using a series of univariate logistic regression models. In a series of adjusted logistic regression models, the exposure-outcome relationships will be adjusted for covariates including age at start of pregnancy, pre-gravid BMI, ethnicity, smoking status, and a disease risk score (DRS) to obtain adjusted odds ratios

with 95% confidence intervals. DRS will be generated for each of the outcomes using logistic regression models considering the outcome as a dependent variable and a range of pre-existing long-term conditions as independent variables. This is done to limit the effect of confounding attributable to prescriptions issued in order to manage the underlying long-term conditions. Relative decrease in p-value (p-RD) prior to and after adjustment for covariates will be calculated using the formula below.(16)

$$p - RD = \frac{p\ (before\ adj\ for\ DRS) - p(after\ adj\ for\ DRS)}{p\ (before\ adjustment)}$$

In addition to statistical measures of disproportionality, descriptive data stratified by their outcome status will be presented. These include numbers and proportions of eligible pregnancies with a prescription of the individual medication or medication combinations (pairs) during the two separate exposure windows.

Benjamini–Hochberg correction for multiple testing will be applied with a threshold of 0.20.

The analyses in each of the four datasets will be conducted separately. Signals arising from each of the four data sources will be reviewed side-by-side through a systematic signal review workshop described below.

The methods in this protocol are reported in line with RECORD (REporting of studies Conducted using Observational Routinely-collected health Data) guidelines [Supplementary Table 2].

Systematic Signal review

A multidisciplinary expert team comprising of epidemiologists, general practitioners, obstetricians, obstetric physicians, pharmacists, data scientists, experts in genetics, internal medicine and pharmacovigilance, and researchers with expertise on the outcome of interest will be invited to a signal detection workshop. The workshop is aimed at collaboratively working and screening potential signals to identify and exclude signals that are likely to be affected by bias and confounding, leaving signals suggestive of causal associations to be further evaluated in future studies.

A list of all the exposure-outcome pairs, the adjusted and unadjusted OR along with 95% confidence intervals, and p-RD will be presented to the multidisciplinary team in the order of statistical significance and strength of association. Exposure-outcome pairs with a clinically significant strength of association without statistical significance will also be provided to the review team to avoid false negatives.

A checklist with the following items will be provided to the review team: (1) confirmation of exposure – outcome temporality to limit the possibility of reverse causality,(30) (2) consideration of concomitant medications to limit the possibility of co-prescription bias,(31) (3) consideration medical history, (4) consideration of demographic features, and (5) consideration of underlying disease and other alternative explanations to limit the possibility of confounding. After consideration of the checklist items, we will request each reviewer to mark the exposure-outcome pairs with three possible response options: (1) “already established”, (2) “warrant further investigation” and, (3) “dismissed”, with reasons for each response. In a group discussion, conflicting responses will be discussed, and a consensus will be made.

For peer review only

Patient and Public Involvement

Patient and public involvement (PPI) have been extensive from design to dissemination of research outputs from the MuM-PreDiCT group. Our PPI representatives have provided advice on the importance and relevance of this study and helped shape the research question. PPI representatives were also involved in screening and identifying the pregnancy outcomes of interest that are relevant to this study. One of the PPI representatives (NM) also co-authored this protocol. PPI representatives will be involved in the interpretation of the results in the future.

Ethics approval and consent to participate

CPRD has ethics approval from the Health Research Authority to support research using anonymised patient data. Use of CPRD and linked HES data for this study is approved by the Independent Scientific Advisory Committee. Use of SAIL databank for this study is approved by the SAIL Information Governance Review Panel. Use of SMR data for this study is approved by the School of Medicine Ethics Committee, acting on behalf of the University of St. Andrews Teaching and Research Ethics Committee. SAIL and SMR data will be analysed within a Safe Haven Research environment. Use of NIMATS and EPS data for this study is approved by the Office for Research Ethics Committees Northern Ireland (ORECNI) and the Honest Broker Governance Board.

Consent for publication

As the study data will be de-identified, consent for publication is not required.

Competing interests

AS has no declarations during the study period; after the study was completed, she has left the University of Birmingham and taken a post in AstraZeneca. The other authors declare no competing interests.

Funding statement

This work is independent research funded by the Strategic Priority Fund "Tackling multimorbidity at scale" programme (grant number MR/W014432/1) delivered by the Medical Research Council and the National Institute for Health Research in partnership with the Economic and Social Research Council and in collaboration with the Engineering and Physical Sciences Research Council. The views expressed are those of the author and not necessarily those of the funders, the NIHR or the UK Department of Health and Social Care. The funders had no role in study design, data collection and analysis, decision to publish, or preparation of the manuscript.

Contributorship statement

Our authors list includes PPI co-investigator NM. AS, SIL, KP, AA-L, CD-M, CM, DOR, HH, JIK, KMA, K-AE, MB, ML, NM, SB, PB, HD, CNP and KN conceived the study and

For peer review only

contributed to the study design. AS led the development of the protocol and drafted the initial manuscript with contribution from SIL, SPBHS, SW, KP, MS, JW, LK and JIK, and supervision from AA-L, CY, CD-M, CM, DOR, KMA, MB, ML, SB, PB, HD, CNP and KN. AAL, NC, AFF, MU, CDM, LK, GS, JIK and MM provided advise on the real-world data sources and their feasibility for use in this study. All authors critically reviewed and revised the protocol drafts and agreed on the final draft manuscript for submission.

For peer review only

Table 1: Scoping real world data source from England to check its' feasibility for signal detection

Data	Features of the dataset	
CPRD	**Setting**	Primary care practices using the Vision® software system
	**Geographical region**	All four nations of the UK, with relatively few practices heavily concentrated in three conurbations and the South
	**Coverage**	4% of the UK
	**Linkage availability to**	• Pregnancy Register and Mother-Baby Linked data<br>• Deprivation data (Patient postcode linked deprivation measures, Practice postcode linked deprivation measures, Index of Multiple Deprivation (IMD), Townsend Deprivation Index, Carstairs Index, Rural-Urban Classification)<br>• Office of National Statistics (ONS) Death Registration Data<br>• Hospital Episode Statistics (HES)<br>• Cancer Registration Data (However, this is not accessed for this study)<br>• Congenital anomaly register (However, this is not accessed for this study)
	**Data content (with linkage)**	• Patient demographics<br>• Consultation details<br>• Symptoms, signs and diagnoses<br>• Referrals to external care<br>• Immunisation details<br>• Records of test data in the GP system<br>• All prescriptions (medications and appliances) issued by the GP
	**Period of data availability**	January 2000 to July 2022
	**Feasibility to identify pregnancy episodes?**	Yes, using CPRD Pregnancy Register

For peer review only

	**Sample size – number of pregnancy episodes**	1.5 million pregnancies
	**Definition of pregnancy start date**	As derived within the CPRD Pregnancy Register
	**Feasibility to identify exposures?**	Yes, using prescription records on the GP system. This is coded using the Dictionary of Medicines and Devices (DM+D). Each product within the dictionary is associated with a BNF code to which the product belongs. The range of exposures for this study will be defined using the BNF code.<br><br>Limitation: The data relates to primary care prescribing only. Secondary care prescribing data, over the counter medications, and data on adherence to treatments are unavailable using this data source. Furthermore, absence of dispensing data may cause exposure misclassification in the instance of prescriptions that are issued but not dispensed
	**Feasibility to identify pregnancy outcomes?**	Yes, using Read code diagnoses within primary care or ICD-10 diagnoses within linked HES data.

For peer review only

Table 2: Scoping real world data source from Wales to check its' feasibility for signal detection

Data	Features of the dataset	
SAIL	Setting	A population level database in Wales. SAIL is a repository of anonymized health and socio-economic administrative data that provides linkage at an individual level using Trusted Third Party (TTP) in the NHS Wales Informatics Service (NWIS).
	Geographical region	Wales
	Coverage	The database contains 90% of all the GP data in Wales and 100% of all hospital admissions (PEDW)
	Linkage availability to	• Primary care data (from Wales Longitudinal General Practice (WLGP))<br>• Secondary care data (from inpatient hospital admissions, inpatient from Patient Episode Database for Wales (PEDW) and outpatient from Outpatient Database for Wales (OPDW)<br>• The National Community Child Health (NCCH)<br>• The Maternal Indicators (MIDS)<br>• The Welsh Dispensing Data Set (WDDS)<br>• The Welsh Demographic Service Dataset (WDSD)<br>• Office of National Statistics (ONS) Annual District Birth Extract<br>• Office of National Statistics (ONS) Annual District Death Extract<br>• Congenital Anomaly Register and Information Service (CARIS)
	Data content (with linkage)	• (WLGP): Signs, symptoms, test results, diagnoses, prescribed treatment, referrals for specialist treatment<br>• (PEDW): attendance and clinical information, diagnoses and operations performed. (OPDW): attendance information for all NHS Wales hospital outpatient appointments<br>• (NCCHD): Comprises information pertaining to birth registration, monitoring of child health examinations, and immunisations.<br>• (MIDS): contains data relating to the woman at initial assessment and to the mother and baby (or babies) for all births.

		• (WDDS): containing information on GP prescribed medications, dispensed medications by community contractors have been linked to the SAIL databank.<br>• (WDSD): used to extract Lower-layer Super Output Area (LSOA) version 2011 information associated with area-level deprivation (the Welsh Index for Multiple Deprivation (WIMD) version 2019)<br>• All births in Wales collected from birth registrations.<br>• All deaths relating to Welsh residents, including those that died out of Wales<br>Information about foetuses or babies who has or is suspected of having a congenital anomaly,
	**Period of data availability**	2000-2022
	**Feasibility to identify pregnancy episodes?**	Yes, using GP, PEDW, NCCHD, and MIDS datasets
	**Sample size – number of pregnancy episodes**	~475,000 pregnancies (from the year 2000 onwards)
	**Definition of pregnancy start date**	Pregnant women can be identified as any woman who have pregnancy codes in the WLGP data, in hospital admissions (PEDW) for pregnancy, or mothers in the NCCH or MIDS data with the baby birth date (Pregnancy end date) and gestational age at birth available.
	**Feasibility to identify exposures?**	Yes, data covers prescriptions that are prescribed in Wales by GP's (general medical practitioners) and non-medical prescribers that have prescribed on behalf of the GP practice, that are then dispensed in the community within Wales. The data includes all prescribed medicines, dressings and appliances that are dispensed each month. Information includes - the number of prescription items dispensed by each community pharmacy in Wales, broken down by the GP practice in which they are prescribed, and also the number of prescription items prescribed in each GP practice in Wales broken down by the pharmacy that dispensed those items.<br><br>Limitation - The data relates to primary care prescribing only. Secondary care prescribing data, over the counter medications, and data on adherence to treatments are unavailable using this data source
	**Feasibility to identify pregnancy outcomes?**	Yes, using Read code diagnoses within primary care or ICD-10 diagnoses within hospital admissions data

For peer review only

Table 3: Scoping real world data source from Scotland to check its' feasibility for signal detection

Data	Features of the dataset	
SMR	**Setting**	Scottish Morbidity Records (SMR) from secondary care data
	**Geographical region**	Scotland
	**Coverage**	All obstetric inpatients and day cases from maternity hospitals in Scotland
	**Linkage availability to**	• Community Prescriptions
	**Data content (with linkage)**	• SMR00 - Outpatients<br>• SMR01 – Inpatient and day cases<br>• SMR02 - Maternity<br>• SMR04 – Mental Health<br>• SMR06 – Cancer<br>• Accident & Emergency<br>• NRS Deaths<br>• Prescribing Information System (community)<br>• Scottish Birth Record (SBR)<br>• National Records of Scotland (NRS) Infant Deaths<br>• NRS Stillbirths<br>• Scottish Linked Congenital Condition Dataset (SLiCCD) (However, this is not accessed for this study)
	**Period of data availability**	SMR02 (pregnancy identification) from 2008-2021 with historical data from women<br>SMR01, SMR00, PIS and A&E database for the first 5 years of life on the children
	**Feasibility to identify pregnancy episodes?**	Yes
	**Sample size – number of pregnancy episodes**	670, 811 births + pregnancies not ending in births
	**Definition of pregnancy start date**	Based on gestational age at booking appointment and or outcome of pregnancy

For peer review only

	**Feasibility to identify exposures?**	Yes, using prescription from the community. This is coded using the Dictionary of Medicines and Devices (DM+D). Each product within the dictionary is associated with a BNF code to which the product belongs. The range of exposures for this study will be defined using the BNF code.<br><br>Limitation: The data relates to primary care prescribing only. Secondary care prescribing data, over the counter medications, and data on adherence to treatments are unavailable using this data source
	**Feasibility to identify pregnancy outcomes?**	Only secondary care

For peer review only

Table 4: Scoping real world data source from Northern Ireland to check its' feasibility for signal detection

Data	Features of the dataset	
NIMATS	**Setting**	NIMATs is available in all five Health Trust areas across Northern Ireland (NI), within each hospital providing maternity services (11 hospitals in total). Access to NIMATS is also available to midwives/clerical staff in various community clinics across NI to allow for booking appointments to be recorded.
	**Geographical region**	Northern Ireland
	**Coverage**	100% within Northern Ireland; full coverage from 2011
	**Linkage availability to**	• NHAIS (Health Register) –<br>○ GP Patient Registration Index<br>○ Household characteristics (e.g. single person households)<br>○ Linkage to GRO (– Maternal deaths; Infant deaths (may be under-represented due to inability to link some infants to health records via Health & Care Number));<br>○ Via postcode,<br>○ NI Multiple Deprivation Measure (NIMDM) – area level deprivation, usually supplied as quintile or decile<br>○ Settlement Band Classification for urban/rural classification (2005-2014) (Post-code used only by HBS staff to aid linkage; not available to researchers)<br>○ Land & Property Services – rateable value of property (another measure of deprivation)<br>• Enhanced Prescribing Database (EPD) - database includes information on all medications that have been prescribed by General Practitioners and dispensed to patients in NI.<br>• Secondary care data – includes hospital episodes from The Patient Administration System (PAS) and ED attendance data.<br>• NI Cerebral Palsy Register

For peer review only - http://bmjopen.bmj.com/site/about/guidelines.xhtml

	**Data content (with linkage)**	• Demographic information<br>• Clinical information on mothers, pregnancies and infants – delivery, investigations, medications, hospital stays, postnatal complications<br>• Mother's past medical and obstetric history.
	**Period of data availability**	2011-2022
	**Feasibility to identify pregnancy episodes?**	Yes. the mother is identified by her unique Health & Care Number, but she has a different hospital maternity number for each pregnancy.
	**Sample size – number of pregnancy episodes**	~300,000 pregnancies
	**Definition of pregnancy start date**	Can be derived from NIMATS variable EDC based on ultrasound scan
	**Feasibility to identify exposures?**	Linkage to EPD; NIMATS also records medications taken at booking interview (maternal self-report), however this is a free-text variable and prone to variations in spelling. It is also deemed potentially disclosive and therefore not released to the researcher. Upon request, HBS may be able to provide a "dummy" variable for medications of particular interest.<br><br>Limitation: The data relates to primary care prescribing only. Secondary care prescribing data, over the counter medications, and data on adherence to treatments are unavailable using this data source.
	**Feasibility to identify pregnancy outcomes?**	Recorded in NIMATS

For peer review only

Table 5: List of pregnancy outcomes included in this signal detection study from the core outcome set published by the ConcePTION and MuM-PreDiCT consortium

List of core outcomes from ConcePTION consortium	Source of outcome documentation			
	**CPRD**	**SAIL**	**NIMATS**	**SMR**
Miscarriage	Data available as variable in CPRD Pregnancy Register	Data unavailable<br>Currently, within SAIL researchers are unable to account for terminations as these are classed as sensitive data and not currently accessible for research purposes	Variable recorded in NIMATS, but only if it has occurred after booking appointment (early miscarriages may be under-represented)	Data available in SMR02 when women are admitted to secondary care
Intrauterine death / Stillbirth / perinatal death	Data available as variable in CPRD Pregnancy Register and linked HES data	Data available as stillbirths flagged in NCCH and Annual District Birth Extract (ABDE) datasets	IUD = Yes<br>Stillbirth = Yes<br>Perinatal death = partial (some infant deaths will not be captured in NIMATS but may be picked up in General Register Office (GRO) or (Patient Administration System) PAS data	Data available in SMR02
Small for gestational age (SGA)	Birthweight, baby's sex and gestational age data available within CPRD Pregnancy Register and linked HES data, from which SGA can be derived	Can be derived from birthweight data available in ADBE and NCCH, gestational age available in the latter	Yes – Variable "Birth Centile" available 2016 onwards<br>If missing, can be derived from NIMATS variables (birthweight, sex and gestational age at delivery)	Can be derived from variables recorded in SMR02 (birthweight, sex and gestational age at delivery)
Preterm Birth	Gestational age available within CPRD Pregnancy Register from which preterm birth can be derived	Data available in NCCH	Can be derived from variables recorded in NIMATS (gestational age at delivery, EDC)	Can be derived from variables recorded in SMR02 (gestational age at delivery, EDC)
Overall Congenital anomalies (CA) including termination of pregnancy due to fetal anomaly	Data available in primary care data, and linkage to baby records identified from mother-baby linked (MBL) data	Data available from the Congenital Anomaly Register and Information Service (CARIS) database and the NCCH_SIG_COND dataset which is used to inform it.	Yes – NIMATS (variable =infant complications; value = "congenital abnormality")	Data unavailable
Specific major congenital anomalies (not including termination of pregnancy due to fetal anomaly)	Congenital anomalies recorded with sufficient quality will be included. Data available in primary care data, and linkage to baby records identified from mother-baby linked (MBL) data	Data available in CARIS database	Data unavailable.<br>Data in hospital discharge records are not suitable for identifying specific congenital anomalies.	Data unavailable
Maternal death	Data available in primary care data	Data available from GP/Patient Episode Data for Wales (PEDW)/ADDE for death during childbirth	Data available in GRO	Data available in National Records of Scotland
**Additional outcomes**				

For peer review only

**from MuM-PreDiCT consortium**				
**Maternal outcomes**				
**Clinical: antenatal**				
Pre-eclampsia, eclampsia, HELLP syndrome, gestational hypertension	Data available in primary care and linked HES data	Data available in primary care and linked PEDW data (if diagnosed)	Data available in the Delivery details description, Present Pregnancy Problems, and Induction reason variables in NIMATS, as well as diagnoses variable in Patient Administration system (PAS)	Recorded from chapter XV of ICD10 Pregnancy, Childbirth and the Puerperium, (O00-O99).
Placenta abruption	Data available in primary care and linked HES data	Data available in primary care and linked PEDW data (if diagnosed)	Data available in the Delivery details description, Indication for Caesarean section variables in NIMATS, as well as diagnoses variable in Patient Administration system (PAS)	Recorded from chapter XV of ICD10 Pregnancy, Childbirth and the Puerperium, (O00-O99).
Venous thromboembolism	Data available in primary care and linked HES data	Data available in primary care and linked PEDW data (if diagnosed)	Data available as postnatal complications variable in NIMATS and as a diagnoses variable in Patient Administration system (PAS)	Recorded from chapter XV of ICD10 Pregnancy, Childbirth and the Puerperium, (O00-O99).
**Clinical: peripartum**				
Preterm premature rupture of membrane (PPROM)	Data available in linked HES data	Data available in primary care and linked PEDW data (if recorded)	Data available as postnatal complications variable in NIMATS and as a diagnoses variable in Patient Administration system (PAS)	Recorded from chapter XV of ICD10 Pregnancy, Childbirth and the Puerperium, (O00-O99).
Severe maternal morbidity	Specific maternal morbidities recorded with sufficient quality will be included. Data available from primary care data	Data available in primary care and linked PEDW data (if recorded)	Data available as specific maternal morbidities in NIMATS	Recorded from chapter XV of ICD10 Pregnancy, Childbirth and the Puerperium, (O00-O99).
Postpartum haemorrhage (PPH)	Data available in linked HES data	Data available in primary care and linked PEDW data (if recorded)	Data available as postnatal complications variable in NIMATS and as a diagnoses variable in Patient Administration system (PAS)	Recorded from chapter XV of ICD10 Pregnancy, Childbirth and the Puerperium, (O00-O99).
**Mental health**				
Self-harm/suicide	Data available in primary care and linked HES data	Data available in primary care, linked PEDW, and emergency admissions data	Data available in Self Harm and Suicide Ideation Register	Data available in National Records of Scotland
Postpartum mental illness	Data available in primary care and linked HES data	Data available in primary care and linked PEDW data (if diagnosed)	Data available as postnatal complications variable in NIMATS and as a diagnoses variable in Patient Administration system (PAS)	Recorded from chapter XV of ICD10 Pregnancy, Childbirth and the Puerperium, (O00-O99).
**Children's outcomes**				
Cerebral palsy/Autism/ADHD/Neurodevelopmental outcomes	Data available in primary care data, and linkage to baby records identified from mother-baby linked (MBL) data	Data available in primary and linked PEDW data	Partial data available in NI Cerebral Palsy Register (NICPR)	Data unavailable

For peer review only

References

1. Sundari Ravindran TK, Teerawattananon Y, Tannenbaum C, Vijayasingham L. Making pharmaceutical research and regulation work for women. BMJ. 2020 Oct 27;371.
2. Watson S, Caster O, Rochon PA, den Ruijter H. Reported adverse drug reactions in women and men: Aggregated evidence from globally collected individual case reports during half a century. EClinicalMedicine. 2019 Dec 1;17:100188.
3. Figueroa Gray M, Hsu C, Kiel L, Dublin S. Getting through the day: A pilot qualitative study of U.S. women's experiences making decisions about anti-nausea medication during pregnancy. BMC Pregnancy Childbirth. 2018 Dec 4;18(1):1–8.
4. Ngure K, Trinidad SB, Beima-Sofie K, Kinuthia J, Matemo D, Kimemia G, et al. Perceived Social Influences on Women's Decisions to use Medications not Studied in Pregnancy. A Qualitative Ethical Analysis of Preexposure Prophylaxis Implementation Research in Kenya. J Empir Res Hum Res Ethics. 2021 Jun 16;
5. UNIVERSITYoF BIRMINGHAM The Case for UK Leadership in the Development of Safe, Effective and Accessible Medicines for Use in Pregnancy. 2022;
6. UKTIS [Internet]. [cited 2022 Nov 23]. Available from: http://www.uktis.org/
7. Ren Z, Bremer AA, Pawlyk AC. Drug development research in pregnant and lactating women. Am J Obstet Gynecol. 2021 Jul 1;225(1):33–42.
8. Lynch MM, Squiers LB, Kosa KM, Dolina S, Read JG, Broussard CS, et al. Making Decisions About Medication Use During Pregnancy: Implications for Communication Strategies. Matern Child Health J. 2018 Jan 1;22(1):92.
9. Subramanian A, Azcoaga-Lorenzo A, Anand A, Phillips K, Lee SI, Cockburn N, et al. Polypharmacy during pregnancy and associated risk factors: a retrospective analysis of 577 medication exposures among 1.5 million pregnancies in the UK, 2000-2019. BMC Med. 2023;21(1):21.
10. Rochon PA, Petrovic M, Cherubini A, Onder G, O'Mahony D, Sternberg SA, et al. Polypharmacy, inappropriate prescribing, and deprescribing in older people: through a sex and gender lens. Lancet Heal Longev. 2021 May 1;2(5):e290–300.
11. Prevalence of polypharmacy in pregnancy | SAPC [Internet]. [cited 2022 Aug 23]. Available from: https://sapc.ac.uk/conference/2021/abstract/prevalence-of-polypharmacy-pregnancy_p1-29-2
12. Steinberg JR, Turner BE, Weeks BT, Magnani CJ, Wong BO, Rodriguez F, et al. Analysis of Female Enrollment and Participant Sex by Burden of Disease in US Clinical Trials Between 2000 and 2020. JAMA Netw Open. 2021 Jun 1;4(6):e2113749–e2113749.
13. Kaufman DW, Kelly JP, Rosenberg L, Anderson TE, Mitchell AA. Recent Patterns of Medication Use in the Ambulatory Adult Population of the United States: The Slone Survey. JAMA. 2002 Jan 16;287(3):337–44.
14. Huybrechts KF, Bateman BT, Hernández-Díaz S. Use of real-world evidence from healthcare utilization data to evaluate drug safety during pregnancy. Pharmacoepidemiol Drug Saf. 2019 Jul 1;28(7):906–22.
15. Cavadino A, Sandberg L, Öhman I, Bergvall T, Star K, Dolk H, et al. Signal Detection in EUROmediCAT: Identification and Evaluation of Medication-Congenital Anomaly Associations and Use of VigiBase as a Complementary Source of Reference. Drug Saf. 2021 Jul 1;44(7):765–85.
16. Demailly R, Escolano S, Haramburu F, Tubert-Bitter P, Ahmed I. Identifying Drugs Inducing Prematurity by Mining Claims Data with High-Dimensional Confounder

Score Strategies. Drug Saf. 2020 Jun 1;43(6):549–59.
17. Cederholm S, Hill G, Asiimwe A, Bate A, Bhayat F, Persson Brobert G, et al. Structured Assessment for Prospective Identification of Safety Signals in Electronic Medical Records: Evaluation in the Health Improvement Network. Drug Saf. 2015 Jan 22;38(1):87.
18. Final Report Summary - EUROMEDICAT (EUROmediCAT: Safety of Medication use in Pregnancy in Relation to Risk of Congenital Malformations) | FP7 | CORDIS | European Commission [Internet]. [cited 2022 Nov 24]. Available from: https://cordis.europa.eu/project/id/260598/reporting
19. Herrett E, Gallagher AM, Bhaskaran K, Forbes H, Mathur R, Staa T van, et al. Data Resource Profile: Clinical Practice Research Datalink (CPRD). Int J Epidemiol. 2015 Jun 1;44(3):827–36.
20. CPRD GOLD July 2022 dataset | CPRD [Internet]. [cited 2022 Nov 26]. Available from: https://cprd.com/cprd-gold-july-2022-dataset
21. Minassian C, Williams R, Meeraus WH, Smeeth L, Campbell OMR, Thomas SL. Methods to generate and validate a Pregnancy Register in the UK Clinical Practice Research Datalink primary care database. Pharmacoepidemiol Drug Saf. 2019 Jul 1;28(7):923–33.
22. Jones KH, Ford D V., Thompson S, Lyons RA. A Profile of the SAIL Databank on the UK Secure Research Platform. Int J Popul Data Sci. 2019 Nov 20;4(2).
23. Lee SI, Azcoaga-Lorenzo A, Agrawal U, Kennedy JI, Fagbamigbe AF, Hope H, et al. Epidemiology of pre-existing multimorbidity in pregnant women in the UK in 2018: a population-based cross-sectional study. BMC Pregnancy Childbirth. 2022 Dec 1;22(1):1–15.
24. Given J, Casson K, Dolk H, Loane M. Evaluation of maternal medications and diseases recorded in Northern Ireland Maternity System (NIMATS) database compared to NI Enhanced Prescribing Database (EPD): a data linkage validation study. Int J Popul Data Sci. 2018 Jun 11;3(2).
25. Lee SI, Eastwood KA, Moss N, Azcoaga-Lorenzo A, Subramanian A, Anand A, et al. Protocol for the development of a core outcome set for studies of pregnant women with pre-existing multimorbidity. BMJ Open. 2021 Oct 1;11(10):e044919.
26. Damase-Michel C, Wurst K. Core evidence elements for generating medication safety evidence for pregnancy using population-based data (D1.2). 2021 Jul 5;
27. Welsh Dispensing Dataset (WDDS) [Internet]. [cited 2022 Aug 23]. Available from: https://web.www.healthdatagateway.org/dataset/50ef6443-ed4b-40f9-97fb-1cfd53be6579
28. 12 Weeks Pregnant | Pregnancy | Start for Life [Internet]. [cited 2023 Feb 14]. Available from: https://www.nhs.uk/start4life/pregnancy/week-by-week/1st-trimester/week-12/#anchor-tabs
29. A healthy lifestyle - WHO recommendations [Internet]. [cited 2023 Jul 11]. Available from: https://www.who.int/europe/news-room/fact-sheets/item/a-healthy-lifestyle---who-recommendations
30. Gail MH, Altman DG, Cadarette SM, Collins G, Evans SJW, Sekula P, et al. Design choices for observational studies of the effect of exposure on disease incidence. BMJ Open. 2019 Dec 9;9(12).
31. Avillach P, Salvo F, Thiessard F, Miremont-Salamé G, Fourrier-Reglat A, Haramburu F, et al. Pilot evaluation of an automated method to decrease false-positive signals induced by co-prescriptions in spontaneous reporting databases. Pharmacoepidemiol Drug Saf. 2014 Feb 1;23(2):186–94.

For peer review only

Supplementary Table 1: Operational definition of pregnancy outcomes included in this signal detection study

Outcomes	Operational definition	Observation time window
**Miscarriage**	(1) Diagnostic code in primary care OR<br>(2) Diagnostic code in secondary care OR<br>(3) Database specific flag for miscarriage	Diagnostic code or flag recorded until 30 weeks of pregnancy (includes 6-week lag period for recording)
**Intrauterine death / Stillbirth / perinatal death**	(1) Diagnostic code in primary care OR<br>(2) Diagnostic code in secondary care OR<br>(3) Database specific flag for IUD/Stillbirth/perinatal death	Diagnostic code or flag recorded from 24 weeks of pregnancy until six weeks after delivery date (includes 6-week lag period for recording)
**Small for gestational age (SGA)**	(1) Diagnostic code in primary care OR<br>(2) Diagnostic code in secondary care OR<br>(3) Database specific flag for SGA OR<br>(4) Database specific continuous variable for gestational age, sex and birthweight (indicating birthweight below the 10th percentile for babies of the same gestational age according to the INTERGROWTH-21st birth weight standard)	Diagnostic code or flag recorded +/- 6 weeks from delivery date
**Preterm birth**	(1) Diagnostic code in primary care OR<br>(2) Diagnostic code in secondary care OR<br>(3) Database specific flag for preterm birth OR<br>(4) Database specific continuous variable for gestational age (indicating gestational age between 22 to 37 weeks)<br>(5)	Diagnostic code or flag recorded +/- 6 weeks from delivery date
**Overall Congenital anomalies (CA)**	(1) Diagnostic code in primary care OR<br>(2) Diagnostic /operational code in secondary care OR<br>(3) Database specific flag for congenital anomalies	Diagnostic code or flag recorded in the baby's medical records until 1 year after birth (delivery date)
**Specific major congenital anomalies**	(1) Diagnostic code in primary care OR<br>(2) Diagnostic code in secondary care OR<br>(3) Database specific flag for congenital anomalies	Diagnostic code or flag recorded in the baby's medical records until 1 year after birth (delivery date)
**Maternal death**	(1) Death flag in primary care OR<br>(2) Death flag in the linked death registry	Death flag recorded until 1 year after pregnancy end date

For peer review only

**Pre-eclampsia, eclampsia, HELLP syndrome, gestational hypertension**	(1) Diagnostic code in primary care OR<br>(2) New primary care prescription of antihypertensives during pregnancy OR<br>(3) Diagnostic code in secondary care OR<br>(4) Database specific flag for pre-eclampsia/HELLP syndrome/gestational hypertension<br><br>Exclusion: Patients with a pre-existing record of chronic hypertension or antihypertensives prior to pregnancy or within 20 weeks of pregnancy	Diagnostic code recorded from 24 weeks of pregnancy until six weeks after pregnancy end date (includes 6-week lag period for recording)
**Placenta abruption**	(1) Diagnostic code in primary care OR<br>(2) Diagnostic code in secondary care OR<br>(3) Database specific flag for placenta abruption	Diagnostic code or flag recorded until 6 weeks after pregnancy end date (includes 6-week lag period for recording)
**Venous thromboembolism**	(1) Diagnostic code in primary care OR<br>(2) Diagnostic code in secondary care OR<br>(3) Database specific flag for venous thromboembolism	Diagnostic code or flag recorded until 12 weeks after pregnancy end date (includes 6-week lag period for recording)
**Preterm premature rupture of membrane (PPROM)**	(1) (Diagnostic code for premature rupture of membrane in primary care OR<br>(2) Diagnostic code for premature rupture of membrane in secondary care OR<br>(3) Database specific flag for premature rupture of membrane)<br><br>AND<br><br>Operational definition met for premature birth	Diagnostic code or flag recorded +/- 6 weeks from delivery date
**Severe maternal morbidity**	(1) Diagnostic code in primary care OR<br>(2) Diagnostic /operational code in secondary care for one of the following morbidities<br><br>1. Acute myocardial infarction<br>2. Aneurysm<br>3. Acute Renal Failure<br>4. Adult respiratory distress syndrome<br>5. Amniotic fluid embolism<br>6. Cardiac arrest or ventricular fibrillation<br>7. Disseminated intravascular coagulation<br>8. Eclampsia	Diagnostic code recorded until 3 months after pregnancy end date

For peer review only

	9. Heart failure or arrest during surgery or procedure<br>10. Puerperal cerebrovascular disorders<br>11. Pulmonary oedema or Acute heart failure<br>12. Severe anaesthesia complications<br>13. Sepsis<br>14. Shock<br>15. Sickle cell disease with crisis<br>16. Air and thrombotic embolism<br>17. Conversion of cardiac rhythm<br>18. Blood products transfusion<br>19. Hysterectomy<br>20. Temporary tracheostomy<br>21. Ventilation	
**Postpartum haemorrhage (PPH)**	(1) Diagnostic code in primary care OR<br>(2) Diagnostic /operational code in secondary care	Diagnostic code recorded until 3 months after pregnancy end date
**Self-harm/suicide**	(1) Diagnostic code in primary care OR<br>(2) Diagnostic /operational code in secondary care OR<br>(3) Suicide as cause of death in the linked death registry	Diagnostic code or death flag recorded until 1 year after pregnancy end date
**Postpartum mental illness**	(1) Diagnostic code in primary care OR<br>(2) Diagnostic code in secondary care	Diagnostic code recorded until 1 year after pregnancy end date
**Cerebral palsy/Autism/ADHD/Neurodevelopmental outcomes**	(1) Diagnostic code in primary care OR<br>(2) Diagnostic code in secondary care	Diagnostic code recorded anytime in the baby's medical records

For peer review only

Supplementary Table 2: The RECORD statement – checklist of items, extended from the STROBE statement, that should be reported in observational studies using routinely collected health data.

	Item No.	STROBE items	Location in manuscript where items are reported	RECORD items	Location in manuscript where items are reported
**Title and abstract**					
	1	(a) Indicate the study's design with a commonly used term in the title or the abstract (b) Provide in the abstract an informative and balanced summary of what was done and what was found		RECORD 1.1: The type of data used should be specified in the title or abstract. When possible, the name of the databases used should be included.<br><br>RECORD 1.2: If applicable, the geographic region and timeframe within which the study took place should be reported in the title or abstract.<br><br>RECORD 1.3: If linkage between databases was conducted for the study, this should be clearly stated in the title or abstract.	Multiple databases are planned to be used. This is mentioned in methods and analysis section of the abstract.<br><br>Mentioned in the methods and analysis section of the abstract.<br><br>No linkage involved in the study
**Introduction**					
Background rationale	2	Explain the scientific background and rationale for the investigation being reported			Introduction section, para 4
Objectives	3	State specific objectives, including any prespecified hypotheses			Aims section
**Methods**					
Study Design	4	Present key elements of study design early in the paper			Study design section within

For peer review only

					methods and analysis
Setting	5	Describe the setting, locations, and relevant dates, including periods of recruitment, exposure, follow-up, and data collection			Table 1
Participants	6	*(a) Cohort study* - Give the eligibility criteria, and the sources and methods of selection of participants. Describe methods of follow-up<br>*Case-control study* - Give the eligibility criteria, and the sources and methods of case ascertainment and control selection. Give the rationale for the choice of cases and controls<br>*Cross-sectional study* - Give the eligibility criteria, and the sources and methods of selection of participants<br><br>*(b) Cohort study* - For matched studies, give matching criteria and number of exposed and unexposed<br>*Case-control study* - For matched studies, give matching criteria and the number of controls per case		RECORD 6.1: The methods of study population selection (such as codes or algorithms used to identify subjects) should be listed in detail. If this is not possible, an explanation should be provided.<br><br>RECORD 6.2: Any validation studies of the codes or algorithms used to select the population should be referenced. If validation was conducted for this study and not published elsewhere, detailed methods and results should be provided.<br><br>RECORD 6.3: If the study involved linkage of databases, consider use of a flow diagram or other graphical display to demonstrate the data linkage process, including the number of individuals with linked data at each stage.	Table 1<br><br>Reference number 23<br><br>No linkage
Variables	7	Clearly define all outcomes, exposures, predictors, potential confounders, and effect modifiers. Give diagnostic criteria, if applicable.		RECORD 7.1: A complete list of codes and algorithms used to classify exposures, outcomes, confounders, and effect modifiers should be provided. If these cannot be reported, an explanation should be provided.	This is a signal detection study with a wide range of exposures and outcomes.
Data sources/ measurement	8	For each variable of interest, give sources of data and details			Table 2

For peer review only

		of methods of assessment (measurement). Describe comparability of assessment methods if there is more than one group			
Bias	9	Describe any efforts to address potential sources of bias			Systematic signal review section under methods and analysis
Study size	10	Explain how the study size was arrived at			Table 1
Quantitative variables	11	Explain how quantitative variables were handled in the analyses. If applicable, describe which groupings were chosen, and why			Exposure section, paragraph 2 and covariates section under methods and analysis
Statistical methods	12	(a) Describe all statistical methods, including those used to control for confounding<br><br>(b) Describe any methods used to examine subgroups and interactions<br>(c) Explain how missing data were addressed<br><br>(d) *Cohort study* - If applicable, explain how loss to follow-up was addressed<br>*Case-control study* - If applicable, explain how matching of cases and controls was addressed<br>*Cross-sectional study* - If applicable, describe analytical methods taking account of sampling strategy			(a) Statistical Analysis section, para 2 under methods and analysis<br>(b) No subgroups<br><br>(c) Covariates section under methods and analysis<br>(d) No matching

For peer review only

		(e) Describe any sensitivity analyses			(e) No sensitivity analyses
Data access and cleaning methods		..		RECORD 12.1: Authors should describe the extent to which the investigators had access to the database population used to create the study population.<br><br>RECORD 12.2: Authors should provide information on the data cleaning methods used in the study.	Table 1<br><br>Described elsewhere (Reference 23)
Linkage		..		RECORD 12.3: State whether the study included person-level, institutional-level, or other data linkage across two or more databases. The methods of linkage and methods of linkage quality evaluation should be provided.	No linkage
**Results**					
Participants	13	(a) Report the numbers of individuals at each stage of the study (*e.g.*, numbers potentially eligible, examined for eligibility, confirmed eligible, included in the study, completing follow-up, and analysed)<br>(b) Give reasons for non-participation at each stage.<br>(c) Consider use of a flow diagram		RECORD 13.1: Describe in detail the selection of the persons included in the study (*i.e.*, study population selection) including filtering based on data quality, data availability and linkage. The selection of included persons can be described in the text and/or by means of the study flow diagram.	Study population section under methods and analysis
Descriptive data	14	(a) Give characteristics of study participants (*e.g.*, demographic, clinical, social) and information on exposures and potential confounders<br>(b) Indicate the number of participants with missing data for each variable of interest			This is a protocol paper

For peer review only

		(c) *Cohort study* - summarise follow-up time (*e.g.*, average and total amount)			
Outcome data	15	*Cohort study* - Report numbers of outcome events or summary measures over time<br>*Case-control study* - Report numbers in each exposure category, or summary measures of exposure<br>*Cross-sectional study* - Report numbers of outcome events or summary measures			This is a protocol paper
Main results	16	(a) Give unadjusted estimates and, if applicable, confounder-adjusted estimates and their precision (e.g., 95% confidence interval). Make clear which confounders were adjusted for and why they were included<br>(b) Report category boundaries when continuous variables were categorized<br>(c) If relevant, consider translating estimates of relative risk into absolute risk for a meaningful time period			This is a protocol paper
Other analyses	17	Report other analyses done—e.g., analyses of subgroups and interactions, and sensitivity analyses			This is a protocol paper
**Discussion**					
Key results	18	Summarise key results with reference to study objectives			This is a protocol paper
Limitations	19	Discuss limitations of the study, taking into account sources of potential bias or imprecision.		RECORD 19.1: Discuss the implications of using data that were not created or collected to answer the specific research question(s). Include	Systematic Signal review section under methods and analysis

For peer review only

		Discuss both direction and magnitude of any potential bias		discussion of misclassification bias, unmeasured confounding, missing data, and changing eligibility over time, as they pertain to the study being reported.	
Interpretation	20	Give a cautious overall interpretation of results considering objectives, limitations, multiplicity of analyses, results from similar studies, and other relevant evidence			This is a protocol paper
Generalisability	21	Discuss the generalisability (external validity) of the study results			This is a protocol paper
**Other Information**					
Funding	22	Give the source of funding and the role of the funders for the present study and, if applicable, for the original study on which the present article is based			Funding section
Accessibility of protocol, raw data, and programming code		..		RECORD 22.1: Authors should provide information on how to access any supplemental information such as the study protocol, raw data, or programming code.	This is a protocol paper

*Reference: Benchimol EI, Smeeth L, Guttmann A, Harron K, Moher D, Petersen I, Sørensen HT, von Elm E, Langan SM, the RECORD Working Committee. The REporting of studies Conducted using Observational Routinely-collected health Data (RECORD) Statement. *PLoS Medicine* 2015; in press.

*Checklist is protected under Creative Commons Attribution (CC BY) license.

For peer review only
